# A Roadmap for Functional Structural Variants in the Soybean Genome

**DOI:** 10.1534/g3.114.011551

**Published:** 2014-05-22

**Authors:** Justin E. Anderson, Michael B. Kantar, Thomas Y. Kono, Fengli Fu, Adrian O. Stec, Qijian Song, Perry B. Cregan, James E. Specht, Brian W. Diers, Steven B. Cannon, Leah K. McHale, Robert M. Stupar

**Affiliations:** * Department of Agronomy and Plant Genetics, University of Minnesota, St. Paul, Minnesota 55108; † Department of Botany, University of British Columbia, Vancouver, British Columbia, Canada, V6T 1Z4; ‡ United States Department of Agriculture, Agricultural Research Service, Soybean Genomics and Improvement Lab, Beltsville, Maryland 20705; § Agronomy and Horticulture Department, University of Nebraska, Lincoln, Nebraska 68583; ** Department of Crop Sciences, University of Illinois, Urbana, Illinois 61801; †† United States Department of Agriculture, Agricultural Research Service, Corn Insects and Crop Genetics Research Unit, Ames, Iowa 50011; ‡‡ Department of Horticulture and Crop Science, The Ohio State University, Columbus, Ohio 43210

**Keywords:** *Glycine max*, soybean, structural variation, CNV, nested association mapping

## Abstract

Gene structural variation (SV) has recently emerged as a key genetic mechanism underlying several important phenotypic traits in crop species. We screened a panel of 41 soybean (*Glycine max*) accessions serving as parents in a soybean nested association mapping population for deletions and duplications in more than 53,000 gene models. Array hybridization and whole genome resequencing methods were used as complementary technologies to identify SV in 1528 genes, or approximately 2.8%, of the soybean gene models. Although SV occurs throughout the genome, SV enrichment was noted in families of biotic defense response genes. Among accessions, SV was nearly eightfold less frequent for gene models that have retained paralogs since the last whole genome duplication event, compared with genes that have not retained paralogs. Increases in gene copy number, similar to that described at the *Rhg1* resistance locus, account for approximately one-fourth of the genic SV events. This assessment of soybean SV occurrence presents a target list of genes potentially responsible for rapidly evolving and/or adaptive traits.

Genome-level diversity arises from a wide spectrum of mutational events, from chromosome-level events (*e.g.*, aneuploidy) to single nucleotide polymorphisms (SNPs). Recently, there has been a surge of interest in mid-level types of polymorphism: changes smaller than chromosomal-level differences but substantially larger than SNPs. This structural variation (SV), which is often observed as large deletions or duplications, occurs on a scale from single genes to sizeable multi-genic regions. SV segments are often referred to as copy number variation (CNV) when there is any difference in copy number across genotypes, or as presence–absence variation (PAV) when some genotypes contain the segment while other genotypes are entirely devoid of the chromosomal segment.

Essentially, two types of SV studies have been published in the plant research community. The first type assesses the global pattern of SV throughout the genome, using array comparative genomic hybridization (CGH) or next-generation sequencing (NGS), or a combination of these platforms. This type of study has become increasingly popular in model plant and crop species. Genome-wide SV profiles have been published recently for maize (*Zea mays*) (Swanson-Wagner *et al.* 2010; Chia *et al.* 2012; Hirsch *et al.* 2014), Arabidopsis (Santuari *et al.* 2010; Cao *et al.* 2011), soybean (*Glycine max*) (Lam *et al.* 2010; McHale *et al.* 2012), barley (*Hordeum vulgare L.*) (Muñoz-Amatriaín *et al.* 2013), and sorghum (*Sorghum bicolor* L.) (Zheng *et al.* 2011) in addition to several other species (Zmieńko *et al.* 2014). These studies have been successful at extracting meaningful biology from the global SV patterns but have not attempted to assess the direct impacts of an individual CNV or PAV on a particular plant phenotype.

The second type of plant SV study focuses on the association between specific CNV/PAV within genes that govern a specific trait of interest. Gene CNVs/PAVs have been associated with numerous traits of biological and agricultural importance (Zmieńko *et al.* 2014). Important examples include glyphosate resistance in Palmer amaranth (*Amaranthus palmeri*) (Gaines *et al.* 2010, 2011), boron tolerance and winter hardiness in barley (Sutton *et al.* 2007; Knox *et al.* 2010), seed coat pigmentation and soybean cyst nematode resistance in soybean (Todd and Vodkin 1996; Cook *et al.* 2012), female gamete fitness in potato (*Solanum tuberosum*) (Iovene *et al.* 2013), flavor quality in strawberry (*Fragaria* × *ananassa*) (Chambers *et al.* 2014), dwarfism and flowering time in wheat (*Triticum spp.*) (Pearce *et al.* 2011; Díaz *et al.* 2012; Li *et al.* 2012), submergence tolerance in rice (*Oriza sativa*) (Xu *et al.* 2006), and aluminum tolerance and glume formation in maize (Han *et al.* 2012; Wingen *et al.* 2012; Maron *et al.* 2013). Interestingly, these studies were often initiated as map-based cloning efforts, where the mapped interval was coincident with a causative structural variant. We are not aware of any published studies in which genome-wide SV profiles have been used to identify or facilitate the discovery of a candidate SV influencing a polymorphic plant trait.

Soybean is a self-pollinating species that has experienced genetic bottlenecks during domestication and modern improvement (Hyten *et al.* 2006; Li *et al.* 2013). To assess standing genomic variation in the germplasm, this study performs SV profiling on 41 soybean accessions to identify high confidence genic CNVs/PAVs. These accessions were used as parents to develop a nested association mapping (SoyNAM) population (previously described by Stupar and Specht 2013). This panel was strategically selected for SV profiling because the SoyNAM population is now being evaluated in the Midwestern United States for several important agricultural traits. Therefore, this study serves two distinct purposes: to increase understanding of the contribution of SV to soybean genetic diversity and to report genes impacted by CNV/PAV that might be candidate loci contributing to phenotypic variation in the SoyNAM population.

## Materials and Methods

### Comparative genomic hybridization

“Williams 82_ISU_01” (denoted hereafter as Wm82-ISU-01) is a sub-line of the reference genome soybean (*Glycine max*) cultivar “Williams 82” (Bernard and Cremeens 1988; Haun *et al.* 2011). The stock of “Williams 82” seed containing Wm82-ISU-01 was originally obtained from Dr. Randy Shoemaker (USDA, Agricultural Research Service) at Iowa State University. Wm82-ISU-01 is the nearest known match to the soybean reference genome assembly version 1.0 (Schmutz *et al.* 2010; Haun *et al.* 2011) and therefore was used as the common reference for all the experiments in this study. Seeds for the 41 soybean nested association mapping (NAM) parents were obtained from the University of Nebraska (see Supporting Information, Table S1 for a list of the NAM parents).

Seeds were planted in 4-inch pots individually containing a 50:50 mix of sterilized soil and Metro Mix. Young trifoliate leaves from 3-week-old plants were harvested and immediately frozen in liquid nitrogen. Frozen leaf tissue was powdered with a mortar and pestle in liquid nitrogen. DNA was extracted using the Qiagen Plant DNeasy Mini Kit according to the manufacturer’s protocol. DNA was quantified on a NanoDrop spectrophotometer.

An updated comparative genomic hybridization (CGH) microarray designed and built by Roche NimbleGen was used that includes 1,404,208 probes. The probes were designed based on the Williams 82 reference sequence assembly version 1.0 (Schmutz *et al.* 2010). The probes, which range between 50 and 70 bp, tile the genome at a median spacing of approximately 500 bp. Labeling, hybridization, and scanning for the CGH experiments were performed as previously described (Haun *et al.* 2011; McHale *et al.* 2012). Briefly, Wm82-ISU-01 was used as the Cy5 reference in all hybridizations, whereas the test genotype was labeled with Cy3. The SegMt algorithm in the DEVA software was used to generate the raw data and identify segments. The program parameters were as follows: minimum segment difference = 0.1, minimum segment length (number of probes) = 2, acceptance percentile = 0.99, and number of permutations = 10. Spatial correction and qspline normalization were applied.

The log_2_ ratio between the Cy3 and Cy5 dyes (*i.e.*, the NAM parent genotype compared to the Wm82-ISU-01 reference) was calculated for each probe. Segments of probes were called significant if the mean of the log_2_ ratio was above the upper threshold or below the lower threshold for that given genotype comparison. The lower threshold for each comparison was set at 3 SDs below the log_2_ ratio mean. The upper threshold for each comparison was set at 2 SDs above the log_2_ ratio mean. Thresholds were separately calculated for each genotype comparison. A custom Perl script was used to process the DEVA generated segments for each genotype and recognize segments beyond these thresholds. The determination of thresholds is explained in greater detail in the File S1 and in Table S2. Significant segments found below or above their respective thresholds were initially classified as “DownCNV” and “UpCNV,” respectively. Collectively, these segments were referred to as “CGH Segment CNV.”

Observations of the initial analysis revealed that while DEVA segmental clustering was successful at merging and detecting large CNV regions, it often did not detect smaller (*e.g.*, gene sized) CNV and had occasionally merged such features into nonsignificant segments. This motivated a second methodology for calling significant CNV using individual CGH probes. To do this, the probes within or overlapping genic space were averaged to get a probe-based log_2_ ratio score for each gene. Genes that did not overlap with any probes were assigned the overlapping DEVA segment average or the average score of the nearest two probes. Genes exhibiting average probe log_2_ values above or below the significance thresholds (as defined in the previous paragraph) were classified as “DownCNV” and “UpCNV,” respectively. Collectively, these genes were referred to as “CGH Probe CNV.” Visual displays of the CGH data were generated using Spotfire DecisionSite software.

### Whole genome sequence data

DNA isolation and whole genome sequencing for each of the 41 NAM parent lines was conducted at the USDA facility in Beltsville, Maryland. Approximately 40 freeze-dried seeds of each NAM genotype were ground to a powder with a steel ball using a Retsch MM400 Mixer Mill at 30 hz for 2 min. DNA was extracted from the ground seed tissue using the Qiagen DNEasy Plant DNA isolation kit. The DNA was fragmentased for 25 min at 37° using the New England Biolabs Next dsDNA fragmentase (New England Biolabs, Beverly, MA) and run on an agarose gel for size selection to obtain fragments in the 400-bp 600-bp range. An “A” overhang was added to the ends of the fragments. The end repaired DNA libraries were ligated with the Illumina paired-end sequencing multiplex adapters (Illumina, San Diego, CA). Illumina paired-end libraries were sequenced for 150 bp on an Illumina HiSeq 2000. The reference line Wm82-ISU-01 was sequenced on an Illumina HiSeq 2000 at the University of Minnesota using a paired-end library and 100 bp reads. Before aligning to the reference, the raw reads were cleaned using minimum base quality score Q30. After this cleaning, the NAM “hub” parent, IA3023 (which was mated to each of the other 40 NAM parents), was sequenced to a depth of 31×. Read depth was variable among the remaining 40 NAM parent lines, ranging from approximately 2× to 8× coverage (Table S1). Wm82-ISU-01 was sequenced to a depth of approximately 13×. The cleaned reads were mapped to the reference genome using BWA MEM (Li and Durbin 2009). The alignments were then cleaned by removing reads that failed vendor quality check, that were PCR or optical duplicates, that are not properly paired, and that mapped to multiple positions.

The number of sequence reads uniquely mapped between the start and stop codons of each gene were counted. Genes that had zero reads across all genotypes (including Wm82-ISU-01) were removed from further analyses. To control for scaling issues, genes that exhibited zero reads in Wm82-ISU-01 and more than one read in at least one NAM parent line were analyzed in parallel. Additionally, genes exhibiting reads in Wm82-ISU-01 and zero reads in at least one NAM parent line were flagged as potential DownCNV and also analyzed separately. RPKM (defined as reads mapped per kilobase per million mapped reads) was calculated across genes and genotypes to standardize the variable genotype coverage and gene size. For each gene, the log_2_ ratio of the NAM parent RPKM divided by the Wm82-ISU-01 RPKM was calculated. Using the same methods as described above for CGH analysis, genes with log_2_ ratios 2 SDs above the mean were considered potential UpCNV and log_2_ ratios below 3 SDs from the mean were considered potential DownCNV for each genotype. Collectively, these genes were referred to as “Sequence CNV.”

### Cross-validation of CGH and sequence data to find significant genes

As described above, CGH and re-sequencing analyses provided three lists of putative structural variants associated with genomic regions: “CGH Segment CNV,” “CGH Probe CNV,” and “Sequence CNV.” A subset of genes was identified from these lists for downstream analysis, including the following: genes found within the “CGH Segment CNVs” and genes found on both the “CGH Probe CNV” and “Sequence CNV” lists (Figure S1). For this subset of genes, the sequence-based log_2_ RPKM ratio values were plotted against the CGH-based log_2_ ratios for all 41 NAM parent genotypes. Structural variants were considered cross-validated among the two platforms when the 41 genotypes clearly split into two or more clusters or collectively clustered beyond stated thresholds. See Figure S2 for a methodological flow chart from data type to CNV cross-validated calls.

The UpCNV and DownCNV classifications were subdivided into more specific categories based on the cross-validation analyses. Estimates of gene copy number per genotype were used as the criterion for classifying each gene into one of six categories that were designated as follows: (1) DownCNV/PAV: one copy in Wm82-ISU-01, zero copies in at least one NAM parent, no more than one copy among all 41 NAM parents; (2) UpPAV: zero copies in Wm82-ISU-01, a single group of one or more copies in at least one NAM parent (Wm82-ISU-01 had few or no reads mapped to these genes while at least one NAM parent exhibited numerous such reads skewing the RPKM based estimates); (3) UpPAV and UpCNV: zero copies in Wm82-ISU-01, multiple groups of one or more copies among the NAM parents; (4) UpCNV and DownCNV: one copy in Wm82-ISU-01, zero copies in at least one NAM parent, more than one copy in at least one NAM parent; (5) UpCNV: one copy in Wm82-ISU-01, more than one copy in at least one NAM parent; and (6) Multi-Allelic UpCNV: one copy in Wm82-ISU-01, multiple groups of one or more copies among the NAM parents.

### Enrichment analyses

Individual gene categories were analyzed for enrichment of protein domains. Protein domains were predicted for the longest open reading frame of each *Glycine max* v1.1 gene model (http://www.phytozome.net/soybean) by Pfam, with gathering thresholds defining prediction cutoffs (Finn *et al.* 2010). For simplicity of presentation, significant results from the 11 PFAM models for leucine-rich repeat domain–containing proteins were described as a single PFAM clan (PFAM clan ID: CL00022). Enrichment of predicted protein domains in each gene list was determined by a hypergeometric distribution with adjustment for multiple hypotheses testing by resampling methods implemented with FuncAssociate 2.0 using 10,000 simulations (Berriz *et al.* 2009).

Paralogs retained from the most recent soybean WGD were identified using QUOTA-ALIGN (Tang *et al.* 2011) using parameters “–merge–self–min_size=5–quota=1:1” to merge local synteny blocks, in a genome self-comparison with a minimum block-size of five genes, to find the paralogs from the most recent duplication. This analysis was run using the predicted amino acid sequences of the *Glycine max* v1.1 gene models (Gmax_v1.1_189_peptide.fa; http://www.phytozome.net/soybean) for cv. Williams 82. Initial anchor points (paralog candidates for QUOTA-ALIGN) were calculated using blastp from the NCBI blast+ package. Genes that were called CNV and contained a homeologous pair were noted and frequency was calculated. Statistical analysis was conducted using the R Statistical software package (R Core Team 2013).

### Simulations

Coalescent simulations (Hudson 2002) were used to compare the site frequency spectrum (SFS) for CNV to those expected under a neutral history in a panmictic population. Hudson’s MakeSamples (ms) generates infinite-sites (Kimura 1969) genetic data under a neutral coalescent process, with specified population-scaled per-locus mutation rates, recombination rates, and migration rates. For CNV, however, a peer-acceptable mutational model does not exist for estimating the per-locus mutation rate. There are, however, map-based recombination rates (Du *et al.* 2012) and population-scaled mutation rate estimates based on DNA resequencing data (Hyten *et al.* 2006).

Previously published estimates of the population per-bp mutation rate (θ_W_) (Hyten *et al.* 2006) were used to estimate the effective population of soybeans. This parameter is related to the effective population size by the equation θ_W_ =4N_e_μ, where N_e_ is the effective population size and μ is the per-bp mutation rate. We solved this equation for Ne, using μ∼7×10^−9^ per bp, as previously estimated (Ossowski *et al.* 2010), which yielded an effective population size estimate of 29,642.

A locus was defined as a single CGH segment, which was experimentally found to be approximately 14 kb on average. The loci were treated as independent and nonoverlapping in the simulations. The observed number of CNV events was used to estimate the mutation rate parameter (theta) for the simulations. An estimate of the map-based recombination rate (Du *et al.* 2012) was used for the recombination rate. The cM/Mb recombination rate estimate was converted into a per-locus rate, with a locus consisting of one CGH segment. The per-locus recombination rate was then multiplied by our estimate of the N_e_, yielding a population-scaled recombination parameter of 21.54.

### Site frequency spectra

Development of a reference-based site frequency spectrum (rSFS) required clustering of adjacent CNV and estimating frequency in the population. Development of an Up rSFS used all genes in the UpCNV and Multi-Allelic UpCNV subclasses while the Down rSFS only used the DownCNV/PAV subclass due to the higher confidence and the simplification to a biallelic model. Assuming nearby genic CNV were the result of a single CNV event and using “CGH Segment CNV” calls as a guide, adjacent cross-validated CNV from the mentioned classes were collapsed into segments. Frequency estimates for individual segments required at least one gene in a segment in a genotype to exceed thresholds for both CGH and resequencing-based SV calls. See Table S3 and Table S4 for specific gene segmentation.

A neutral reference-based site frequency spectrum was generated from the simulation output from MS (Hudson 2002). An SFS in the typical fashion could not be constructed because the CGH data are heavily ascertained. That is, the CGH data are an all-by-one comparison rather than a pairwise comparison, as MS creates. Therefore, the first chromosome in the MS output was designated as the “reference” and differences were counted from the reference chromosome. Because “0” denotes the ancestral state (presence) and “1” denotes the derived state (absence), every site that had a “1” in the reference was discarded. The result is that the SFS is built from sites where Wm82 has the “ancestral” state, and the other genotypes have the “derived” state. The neutral simulations and empirical CNV distribution were then compared for only the DownCNV and UpCNV classes. The CNV distributions were based on segments rather than individual genes by analyzing only segments with cross-validated genes within the DownCNV/PAV and UpCNV classes. Segment CNV distributions for the rSFS more properly reflect the mutational model in which CNV likely originate as segments and not gene-by-gene.

## Results

### Genome-wide patterns of structural variation among the soybean NAM parent lines

**Figure 1:**
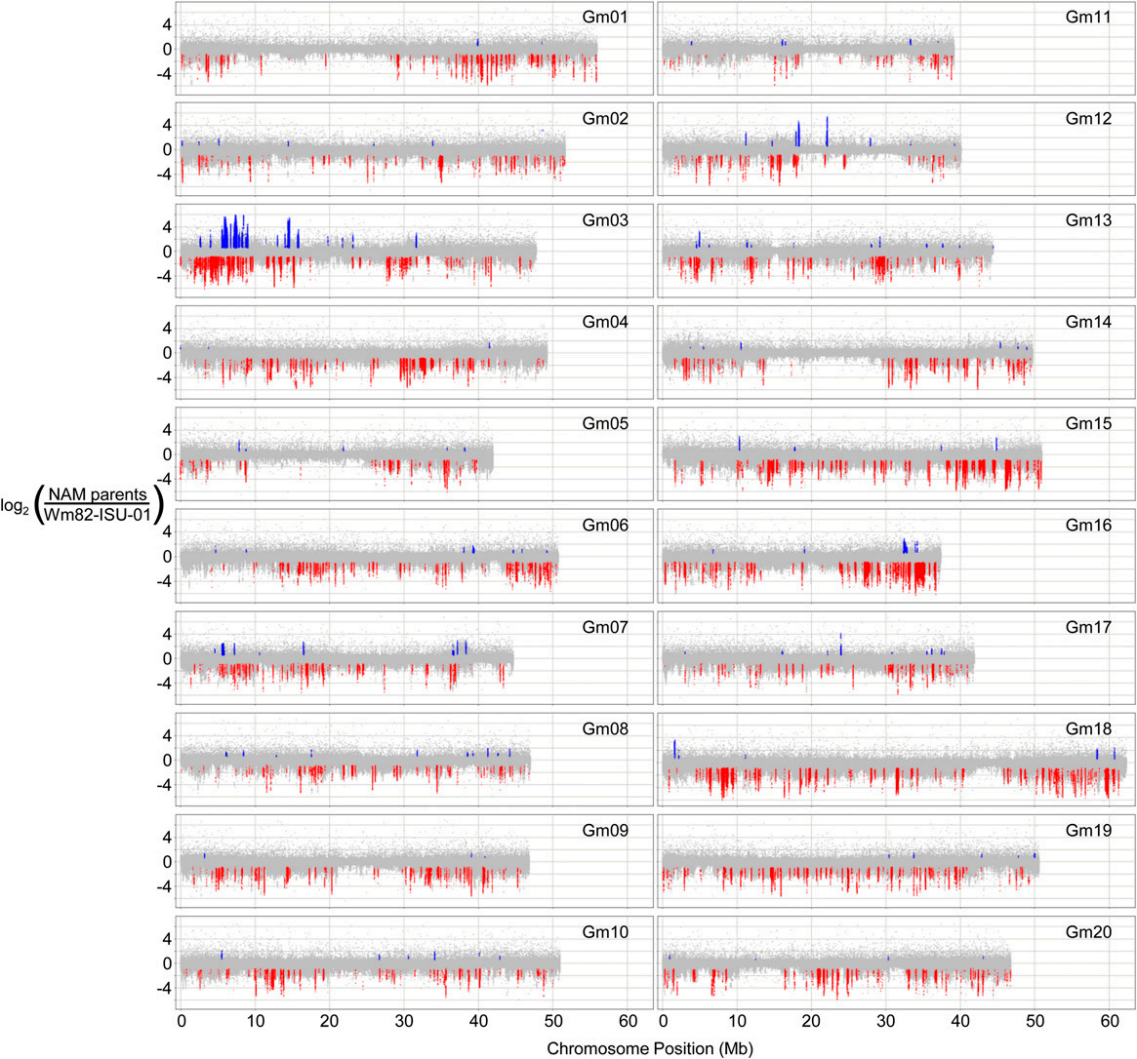
Genome-wide view of copy number variation found in the soybean NAM parents. Data points are the log_2_ ratio of each genotype *vs.* the Williams82-ISU-01 reference for each probe. Colored spots denote probes within segments that exceed threshold: blue for UpCNV and red for DownCNV.

The soybean NAM parents, which include a diverse set of individuals from breeding programs and international introductions, represent a relatively wide sampling of 41 different accessions within maturity groups II–V (Table S1). Initial analyses of deletions and duplications among these soybean NAM parent lines were conducted using a 1.4 million feature comparative genomic hybridization (CGH) tiling microarray platform. Comparative hybridizations were performed between each of the 41 lines (labeled with Cy3 dye) and the reference genome genotype “Wm82-ISU-01” (labeled with Cy5 dye, referred to as “Wm82” henceforth). Figure 1 is an overlay of the 41 CGH comparisons across the 20 chromosomes. Values plotted in red denote genomic segments that are putatively absent in at least one of the 41 NAM parent lines; these were classified as “CGH Down segments.” Blue peaks denote genomic segments that either exhibit copy number gains relative to Wm82 in at least one NAM parent line or are present as a single copy in at least one NAM parent line but are absent in Wm82; these were classified as "CGH Up segments." The CGH analysis identified changes in hybridization intensity contributing to an average of 282 Down and 34 Up segments per NAM parent line relative to Wm82.

Resequencing data on the 41 NAM parent lines and Wm82 were used to cross-validate the CGH segment data and to better estimate the deletion and duplication rates associated with predicted gene models (gene models were based on annotation version 1.1). RPKM values were used to estimate gene copy number from resequencing data. Estimates of gene copy number based on RPKM ratios were compared to those based on the CGH data. Genes with similar copy number estimates in both CGH and resequencing across genotypes were considered “cross-validated” and were included in the downstream analyses. The cross-validated gene set included 339 gene models exclusively associated with Up regions, 1100 gene models exclusively associated with Down regions, and 89 gene models associated with both Up and Down regions among various NAM parents.

Cross-validation between the CGH and resequencing data also identified regions of presumed heterogeneity within some of the 41 NAM parent lines. DNA from approximately 40 plants was bulk-isolated from each line for the resequencing platform, whereas a single individual plant was sampled for the CGH platform. Therefore, some SV genes that reside in regions of intra-cultivar heterogeneity could be identified as exhibiting SV on one platform while matching Wm82 on the other platform. Examples of such heterogeneity are shown in Figure S3, both for a series of genes linked in a PAV region (Figure S3A) and genes exhibiting UpCNV (Figure S3B). Heterogeneity among samples was particularly problematic for lines 4J105-3-4, LD02-4485, LG03-3191, and LG04-4717 (the parents to NAM populations 03, 12, 25, and 26, respectively).

A database was developed to make all the processed CGH and RPKM data publicly available (http://stuparlabcnv.cfans.umn.edu:8080/). Data for all loci are reported, along with scatterplots that compare the CGH and RPKM values.

### Subclassification of SV profiles and identification of potential gain-of-function variants

**Figure 2:**
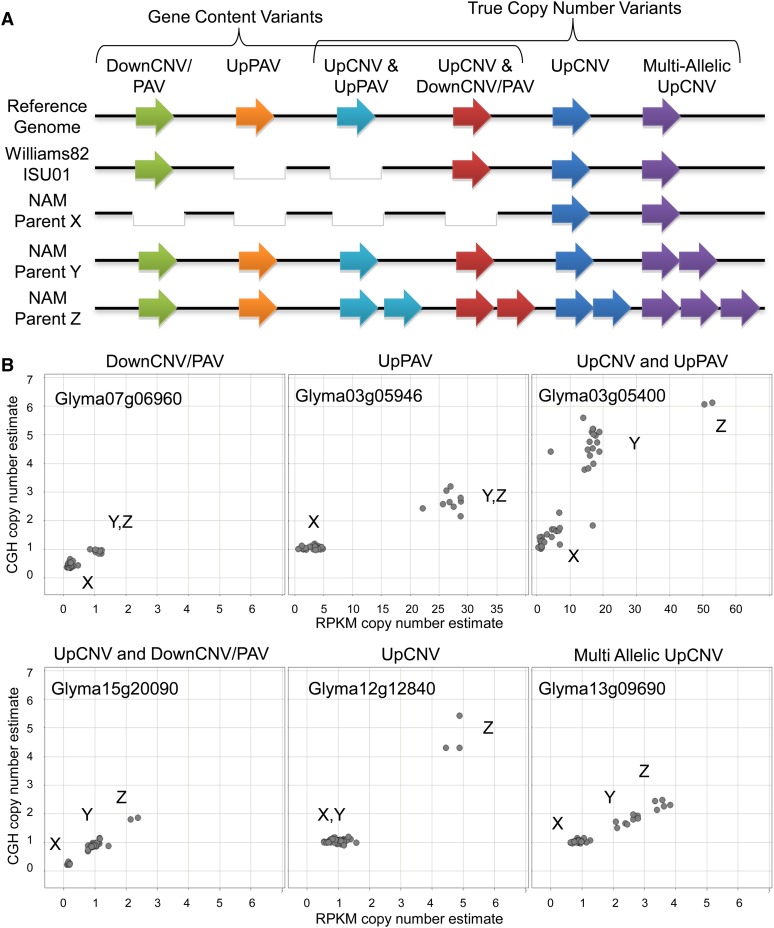
Classification system for CNVs that were associated with gene models. (A) Presence–absence and copy number status for a hypothetical gene in each of the six classes. Genes are found in one of three states: single copy, absent (white gap), or multiple copies (two or more arrows). (B) Gene representatives for each of the six classes showing allelic clusters. Each gene shows one data point for each of the 41 genotypes. The estimated copy number from sequence depth and CGH are shown on the X and Y axes, respectively.

To better describe the range of structural variation observed across the NAM parental lines, each of the cross-validated genes were placed into one of six categories (Figure 2 and Table 1). Down segments, as shown in Figure 1, are referred to as either Down copy number variants (DownCNV) or Down present–absent variants (DownPAV). The simplest interpretation of the CGH data are that many Down structural variants are DownPAV, given that the CGH platform was purposefully designed with probes that have one unique match (one copy) in the “Williams 82” reference genome sequence. Therefore, significant Down segments were not distinguished into subclasses and instead were classified as a single “DownCNV/PAV” category.

**Table 1:** The number of gene models identified within six structural variation categories

	Gene Models Evaluated	DownCNV/DownPAV	UpPAV	UpCNV and UpPAV	UpCNV and DownCNV/PAV	UpCNV	Multi-Allelic UpCNV
Wm82-ISU-01 copy number	1	1	0	0	1	1	1
NAM parent copy number	—	0	1 or >1	>1 and (1 or >>1)	>1 and 0	>1	>1 and >>1
Genes with syntenic paralog	32,464	149	4	1	10	71	9
Genes without syntenic paralog	21,369	951	96	15	79	122	21
Total genes assessed	53,833	1100	100	16	89	193	30

The first two rows indicate the definition of each category based on the observed presence and copy number differences between Wm82-ISU-01 and at least one of the 41 NAM parent lines. The next two rows indicate the number of genes exhibiting each category among the subsets of genes that have maintained a syntenic paralog or have not maintained a syntenic paralog.

Cross-validated Up genes were sorted into the five remaining categories (Figure 2). Any Up genes that were also identified as Down in at least one other NAM parent line were placed into a class designated “UpCNV and DownCNV.” The remaining Up genes were sorted according to their inferred presence–absence status in Wm82-ISU-01 and their mode of copy number distribution among the genotypes (bimodal or polymodal) (Figure 2 and Table 1) (see *Materials and Methods* section for additional details on the classification criteria). Table S5 gives the full list of gene models that were placed into each of the six categories.

Approximately 72% of the 1528 cross-validated genes were placed in the DownCNV/PAV class (Table 1). An additional 205 genes were placed into other “content variant” classes, which are interpreted as being present in some genotypes while being absent in others (Figure 2 and Table 1).

**Figure 3:**
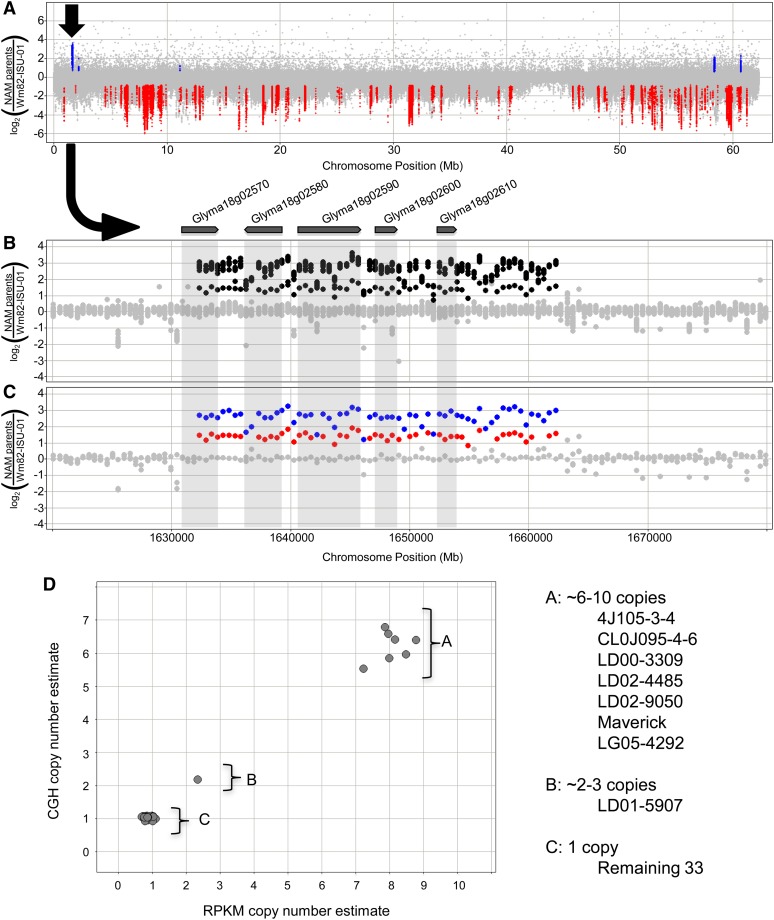
Copy number variation at the soybean cyst nematode locus *Rhg1*. (A) The copy number variant (arrow) is clearly visible from a full view of the chromosome 18 CGH results, overlaying data from all 41 genotypes. (B) The view from (A) is zoomed-in on the 31-kb UpCNV segment that overlaps five gene models (Cook *et al.* 2012). (C) Viewing only one genotype from each allele class confirms a clear separation between three different copy number states. (D) Cross-validation of the CNV for Glyma18g02590 using both CGH (y-axis) and sequence depth (x-axis) analyses.

There were four categories in our classification system that included genes that are duplicated in some genotypes but are not duplicated in Wm82 or other lines. These categories (which all include “UpCNV” in the name) (Figure 2) encompass a total of 328 genes. The five genes located within the soybean cyst nematode resistance QTL *Rhg1* represent a clear example of this type of variation. The variants of the resistant *Rhg1* phenotype have been attributed to the tandem duplication (up to 10-fold) of a 31-kb interval that includes these genes on chromosome 18 (Cook *et al.* 2012, 2014). One copy of this interval, as found in the reference genome of “Williams 82,” is associated with the SCN susceptibility locus (*rhg1*). An allele with three copies of the 31-kb interval has intermediate resistance (*Rhg1-a*), whereas an allele with 10 copies confers the highest known level of resistance (*Rhg1-b*) (Cook *et al.* 2012). Our cross-validated analysis confirmed the presence of at least these three different classes of *Rhg1* copy number among the soybean NAM parents (Figure 3).

**Figure 4:**
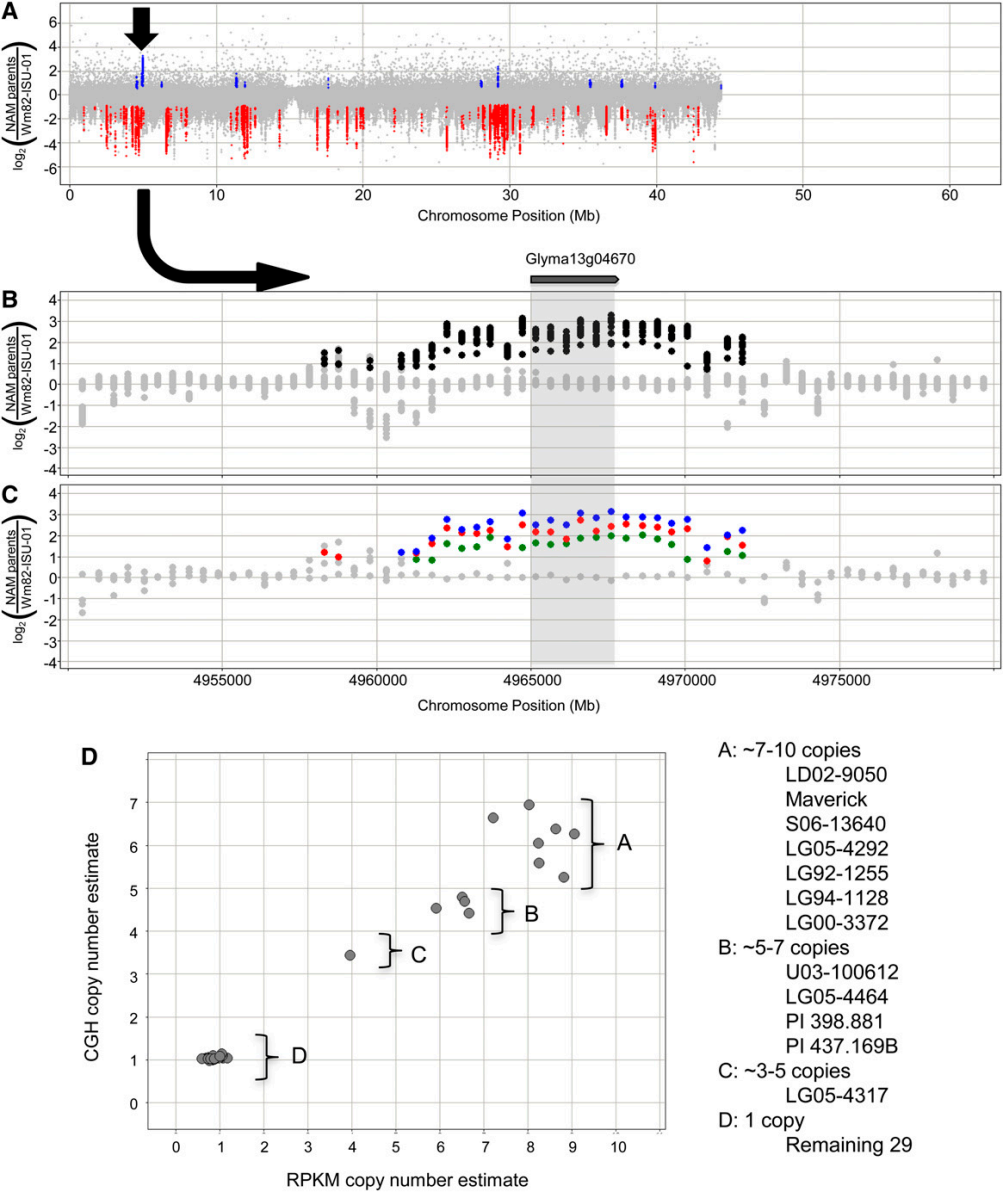
Copy number variation at Glyma13g04670. (A) The copy number variant (arrow) is visible from a full view of the chromosome 13 CGH results, overlaying data from all 41 genotypes. (B) The view from (A) is zoomed in on the approximately 10-kb UpCNV segment that overlaps with Glyma13g04670, revealing multiple CNV classes. (C) Viewing one genotype from each predicted class confirms distinct copy number states. (D) Cross-validation of the CNV for Glyma13g04670 using both CGH (y-axis) and sequence depth (x-axis) analyses, revealing at least four copy number classes.

A small number of gene models exhibited a SV profile similar to *Rhg1*, in which multiple (≥3) copy number classes were observed among the NAM parents. One such example is Glyma13g04670 (named Glyma.13g068800 in the annotation version Wm82.a2.v1), which is embedded within an approximately 10-kb to 15-kb segment on chromosome 13 that exhibits at least four different copy number levels (Figure 4). The Glyma13g04670 gene has been uncharacterized in soybean, but it has been annotated as a Cytochrome P450 with similarity to *Arabidopsis CYP82C4* (Murgia *et al.* 2011). Sequence reads that map to the approximate boundaries of the duplicated approximately 10-kb to 15-kb segment were individually analyzed in genotypes with either one copy or multiple copies of Glyma13g04670. Genotypes with multiple copies of Glyma13g04670 showed reads mapping to chromosome position 4.971 Mb at one end, and then to position 4.958 Mb at the other end (Figure S4). This indicates that the increased copy number of Glyma13g04670 in these genotypes is at least partially caused by a tandem duplication of ∼14-kb interval spanning from position 4.958 Mb to 4.971 Mb on chromosome 13.

### Population analysis and SV enrichment patterns

The lists of genes associated with the six cross-validated structural variation categories were investigated for enrichment within Pfam-predicted protein classes (Finn *et al.* 2010). This analysis indicated an enrichment in the protein domains characteristically encoded by resistance genes (*R*-genes), including leucine-rich repeat (LRR), nucleotide binding (NB), and Toll-interleukin receptor (TIR) protein domains (Table 2) (Kruijt *et al.* 2005; McHale *et al.* 2006). In contrast, enrichment of other protein domains in genes unrelated to disease resistance was not consistently evident among the examined SV categories (Table 2).

**Table 2:** Gene models with specific Pfam domains that are enriched for associations with SV

Pfam ID	Description	Total in Soybean Genome	DownCNV/PAV		UpPAV		UpCNV and UpPAV		UpCNV and DownCNV/PAV		UpCNV		Multi-Allelic UpCNV	
			OBS	EXP	OBS	EXP	OBS	EXP	OBS	EXP	OBS	EXP	OBS	EXP
CL0022	Leucine-rich repeat	1110	168**	23	7	2	3	0	17**	2	2**	4	6	1
PF07714	Protein tyrosine kinase	786	38*	16	0	1	1	0	4	1	3	3	3	0
PF08263	Leucine-rich repeat N-terminal domain	550	74**	11	1	1	0	0	9**	1	10	2	3	0
PF00931	NB-ARC domain	454	112**	9	6	1	6**	0	13**	1	9	2	2	0
PF01582	Toll-interleukin receptor	196	30**	4	3	0	3	0	2	0	0	1	0	0
PF14368	Probable lipid transfer	104	14**	2	0	0	0	0	0	0	0	0	0	0
PF12819	Carbohydrate-binding protein of the ER	95	14**	2	0	0	0	0	2	0	1	0	0	0
PF14111	Domain of unknown function (DUF4283)	82	10*	2	0	0	0	0	2	0	1	0	0	0
PF13947	Wall-associated receptor kinase galacturonan-binding	71	10*	1	0	0	0	0	1	0	2	0	0	0
PF14380	Wall-associated receptor kinase C-terminal	33	10**	1	0	0	0	0	0	0	0	0	0	0
PF05686	Glycosyl transferase family 90	20	7**	0	0	0	0	0	0	0	0	0	0	0
PF05018	Domain of unknown function (DUF667)	7	5**	0	0	0	0	0	0	0	0	0	0	0
PF00499	NADH-ubiquinone/plastoquinone oxidoreductase chain 6	2	0	0	2*	0	0	0	0	0	0	0	0	0

The number of gene models expected to be associated with SV is shown, compared with the number of gene models observed to be associated with SV for each category. Significance of enrichment was determined by the Fisher exact test with a resampling approach to correct for multiple hypotheses as implemented by the FuncAssociate 2.0 (Berriz *et al.* 2009) program using 10,000 simulations (*P < 0.01, **P < 0.001). Only Pfam domains significantly enriched (*P* < 0.01) in at least one SV category were listed.

The next set of analyses focused on the duplicated nature of the soybean genome. Soybean is often referred to as a paleopolyploid, as it retains remnants of whole-genome duplications (WGDs) that occurred approximately 13 million years ago (in the *Glycine* genus), and approximately 59 million years ago (soon after early diversifications in the legume family) (Schmutz *et al.* 2010). An even older genome triplication is also apparent in comparisons of some regions of the soybean genome (Severin *et al.* 2011). Soybean retained a large proportion of duplicate genes from the most recent WGD—with published estimates ranging from ∼43% to 68% of genes retained (Schmutz *et al.* 2010; Severin *et al.* 2011). In our analysis, approximately 60% (32,464/53,833) of the soybean gene models from annotation version 1.1 have retained a syntenic paralog, the majority of which are presumed to be derived from the most recent WGD (Table S6). Genes with retained syntenic paralogs were substantially underrepresented among the gene content variants list (Table 1). Among all categories, SVs were found in only 0.75% (244/32,464) of genes with retained syntenic paralogs, whereas CNVs were found in 6.0% (1284/21,459) of the genes that have not retained a syntenic paralog. This represented an eightfold difference between the two groups of genes. However, this difference was not as severe for the quantitative UpCNV categories (*e.g.*, UpCNV was identified in ∼0.22% of genes with syntenic paralogs and in ∼0.57% of genes without syntenic paralogs) (Table 1).

For genic SV segments, the number of NAM parent lines that exhibited differences compared to Wm82 was analyzed to look for evidence of deviations from a neutral evolution null hypothesis. This analysis included the 117 Up segments (mean of 13,580 bp; median of 3182 bp) and 547 Down segments (mean of 14,958 bp; median of 2775 bp) that overlap with at least one gene identified as CNV/PAV. The frequency of lines showing significant differences compared to Wm82 was calculated for each of these segments. Experimental observations were used as parameters of approximate segment size for simulation of a neutral model under the coalescent. As shown in Figure S5, Down segments closely reflected the frequency spectrum of the simulated neutral model. For Up segments, the frequency spectrum is skewed toward an excess of singleton variants, *i.e.*, those observed only in one NAM parent line (Figure S5).

## Discussion

In this study, we identified genic SV events in the genomes of 41 genetically diverse soybean lines. The observed SV data confirmed major trends previously observed in a smaller analysis of just four soybean accessions. Those trends included an enrichment of SV genes arranged in tandemly duplicated blocks and an association of SV variation with genes contributing to biotic stress responses (McHale *et al.* 2012). Moreover, with the larger dataset obtained in this study, a much more detailed analysis was possible, which provided more definitive evidence for the broader patterns that influence soybean genome diversity, particularly regarding duplicated genes and the distribution of SV frequencies.

Paleopolyploidy is a major defining feature of the soybean genome that experienced two whole genome duplication events approximately 59 and 13 million years ago (Schmutz *et al.* 2010). A majority of soybean genes are present in at least two copies, and a large percentage of these genes have retained duplicates since the most recent genome doubling event. It has been suggested that this feature makes soybean a difficult system for use in functional genomics, because gene redundancy will provide a buffer against the effects of mutagenesis on plant phenotypes. Given the large number of duplicate genes present in soybean, one might expect that the retained duplicates would frequently acquire SV because the loss or functional alteration of duplicate genes may not have a deleterious outcome due to its “backup” copy and, of course, could provide new opportunities for phenotypic plasticity. However, in this study, we found that genes with retained paralogs from the most recent WGD event are underrepresented for associations with SV. This trend was most striking in the PAV events. These findings are likely due in part to enrichment of SV in hypervariable regions, where WGD-derived duplicates may be lost (or not detected) due to local gene cluster expansions and contractions. However, the low rate of SV in regions with retained WGD-derived paralogs also suggests that retention of these duplicate genes may be biologically significant, either due to diversification of biological functions (*e.g.*, neofunctionalization or subfunctionalization) (Roulin *et al.* 2012) or for maintaining proper stoichiometry within regulatory networks (in concordance with the gene balance hypothesis) (Birchler and Veitia 2012). These results coincide with patterns found in mammals and other vertebrates, where preserved WGD-derived paralogs often exhibit low rates of SV across the populations (Makino *et al.* 2013). Taken together, the global trend of SV data in soybean suggests that the “core” set of soybean genes maintained throughout the domesticated germplasm includes a high percentage of ancient homeologous/duplicate genes that have been retained since the most recent polyploidization event. However, experimental biases may also contribute to this observation, because both the CGH platform design and resequencing data analyses require unique sequence tracts to detect a specific gene model; such unique sequences are less abundant among duplicated genes.

A preliminary assessment of SV frequency patterns was conducted by comparing those patterns with a simulated neutral model site frequency for Up and Down genomic segments located within genic regions. The data indicated that UpCNV regions are enriched for rare variants. This stands in contrast to what has been observed at the *Rhg1* locus, where additional copies of a 31-kb segment increases tolerance to soybean cyst nematode (Cook *et al.* 2012). Clearly, haplotypes with increased copies of *Rhg1* are actively being selected by breeding programs. However, there is growing evidence that gene copy number gains may oftentimes be detrimental to fitness (Katju and Bergthorsson 2013).

This poses an interesting question. Can SV profiles be used to predict which copy number changes might provide an adaptive advantage? One could argue that an SV profile of *Rhg1* (Figure 3) may have facilitated the cloning of this locus, as the striking copy number increase for these genes may have immediately established them as candidates located within the genetically mapped interval. Based on the assumption that an increase in copy number confers phenotypic novelty due to altered transcription state, it is reasonable to expect that genes with copy number increases found in multiple genotypes (and at multiple different copy number levels) may be more likely to confer adaptive (and selected) traits, as with *Rhg1* (Cook *et al.* 2012). One such gene from the current study is the cytochrome P450 gene Glyma13g04670, which exhibited a full spectrum of copy number states (up to approximately 10 copies) among the 41 soybean accessions. This is a particularly interesting candidate because there are several published examples of P450 genes acting in biotic and abiotic stress response, as well as herbicide tolerance pathways (Schuler and Werck-Reichhart 2003; Saika *et al.* 2014).

The potential adaptive effect of SV remains largely unexplored. While the association of SV genes in defense gene clusters has long been known (Michelmore and Meyers 1998), there is mounting evidence that copy number gains in specific genes can have tremendous effects on abiotic stress tolerance. Previous studies of barley and maize have specifically identified copy number gains and presence–absence variants that provide enhanced tolerance to stressed soil conditions, such as boron and aluminum toxicity (Sutton *et al.* 2007; Maron *et al.* 2013). Discovery of such loci will become increasingly relevant for the soybean community as crop production expands into poorer soils, or as soils continue to accumulate heavy metals and other chemicals after years of intensive agriculture. The parental CNV and PAV data obtained in these 41 NAM parents will be increasingly useful when the progeny of the NAM parent matings are evaluated for agronomic phenotypes (to be released in May 2015) and potentially stress-related phenotypes in the future.

## Supplementary Material

Supporting Information
