# Supplementary material for: A Roadmap for Functional Structural Variants in the Soybean Genome
Source: G3 (Bethesda). 2014 May 22;4(7):1307–18. doi: 10.1534/g3.114.011551 (PMC4455779; doi:10.1534/g3.114.011551)
Supplement: Supporting Information [file supp_4_7_1307__index.html]

A Roadmap for Functional Structural Variants in the Soybean Genome — Supporting Information 

# A Roadmap for Functional Structural Variants in the Soybean Genome

## Supporting Information for Anderson *et al.*, 2014

**Files in this Data Supplement:**

- Supporting Information - File S1, Figures S1-S5, and Tables S1-S6 (PDF, 410 KB)
- File S1 - Determination of CGH thresholds. (PDF, 115 KB)
- Figure S1 - Venn diagram of the number of significant copy number variant gene models identified by three different detection methods (see Experimental procedures section for descriptions of the three methods). (PDF, 186 KB)
- Figure S2 - Methodological flow chart of the two data types and three different methods used in this analysis. (PDF, 184 KB)
- Figure S3 - Presumed intra-cultivar heterogeneity results in incongruity between resequencing (x-axis) and CGH (y-axis) copy number estimates for some gene x cultivar comparisons. (PDF, 186 KB)
- Figure S4 - Sequence-based evidence for tandem repeats at a ~14-kb interval containing Glyma13g04670. (PDF, 119 KB)
- Figure S5 - Reference based site frequency spectrum for UpCNV (A) and DownCNV (B) compared with simulated neutral frequencies. (PDF, 157 KB)
- Table S1 - Origin, maturity grouping and sequence depth of the soybean NAM parental genotypes assessed in this study (adapted from Stupar and Specht, 2013). (PDF, 113 KB)
- Table S2 - Repeatability of technical replications at variable thresholds. (PDF, 112 KB)
- Table S3 - Segmentation and Frequency of Up rSFS. (.xlsx, 22 KB)
- Table S4 - Segmentation and Frequency of Down rSFS. (.xlsx, 64 KB)
- Table S5 - Genes present in Cross-validated classes. (.xlsx, 31 KB)
- Table S6 - Paralogous gene pairs in the soybean genome derived from ancient whole-genome duplication(s). (.xlsx, 390 KB)
