# Supplementary material for: A Roadmap for Functional Structural Variants in the Soybean Genome
Source: G3 (Bethesda). 2014 May 22;4(7):1307–18. doi: 10.1534/g3.114.011551 (PMC4455779; doi:10.1534/g3.114.011551)
Supplement: Supporting Information [file supp_g3.114.011551_011551SI.pdf]

G3: Genes | Genomes | Genetics

A roadmap for functional structural variants in the soybean genome

Justin E. Anderson[*], Michael B. Kantar[*, §], Thomas Y. Kono[*], Fengli Fu[*], Adrian O. Stec[*], Qijian Song[†], Perry B. Cregan[†], James E. Specht[‡], Brian W. Diers[**], Steven B. Cannon[§§], Leah K. McHale[††], and Robert M. Stupar[*,1]

[*] Department of Agronomy and Plant Genetics, University of Minnesota, St. Paul, MN 55108
[§] Department of Botany, University of British Columbia, Vancouver, BC, Canada, V6T 1Z4
[†] USDA, Agricultural Research Service, Soybean Genomics and Improvement Lab, Beltsville, MD 20705
[‡] Agronomy & Horticulture Department, University of Nebraska, Lincoln, NE 68583
[**] Department of Crop Sciences, University of Illinois, Urbana, IL 61801
[§§] USDA, Agricultural Research Service, Corn Insects and Crop Genetics Research Unit, Ames, IA 50011
[††] Department of Horticulture and Crop Science, The Ohio State University, Columbus, OH 43210

[1]Author for correspondence:
Robert M. Stupar
University of Minnesota
1991 Upper Buford Circle
411 Borlaug Hall
St. Paul, MN 55108-6026
Office: 612-625-5769
Fax: 612-625-1268
Email: rstupar@umn.edu

All comparative genomic hybridization data in this study can be found as accession number GSE56351 in the National Center for Biotechnology Information Gene Expression Omnibus (http://www.ncbi.nlm.nih.gov/geo). NCBI accession numbers for all resequencing data will added in the final proof.

DOI: 10.1534/g3.114.011551

File S1

Determination of CGH thresholds

Probes on the microarray were designed to bind at unique single copy sequences in the reference genome. This assumption influenced the method used to determine significant segments in the CGH data. Applying this assumption, significant DownCNV should often represent sequences that are present as a single copy in Wm82-ISU-01, but are absent in the test genotype. However, it is also possible that hybridization differences can be caused by present but highly polymorphic sequences, which may also reduce the Cy3 signal from the test genotype. True PAV segments, in contrast, should exhibit a stronger log_2 ratio reduction, as the denominator in the calculation will be expected to be nearly zero. Therefore, we applied a stringent threshold of three standard deviations to buffer against the detection of polymorphic sequences, and enrich the percentage of true PAV among the Down calls. Analysis of technical replicates of the IA3023 versus Wm82-ISU-01 comparison confirmed the highest level of repeatability using this threshold (Supplemental Table 4).

UpCNV threshold determination required a different set of assumptions. Again, segments were expected to be present as a single copy in Wm82-ISU-01. Furthermore, segments that were absent in Wm82-ISU-01 and present in the test genotype will exhibit large log_2 ratio values that will most certainly exceed the threshold. Instead, the challenge is to detect the quantitative variants that are present as a single copy in Wm82-ISU-01 but present in two or three copies in the test genotype. The *Rhg1* locus, which harbors a well-defined copy number increase across a 31.2-kb interval on chromosome 18 (Cook *et al.* 2012), was used to empirically determine an appropriate threshold to accurately call UpCNV. It was determined that a threshold of two standard deviations above the mean was capable of detecting the 3-copy haplotype of *Rhg1*, whereas a three standard deviation threshold was not. Therefore, the two standard deviation threshold was applied across the samples for the detection of UpCNV segments.

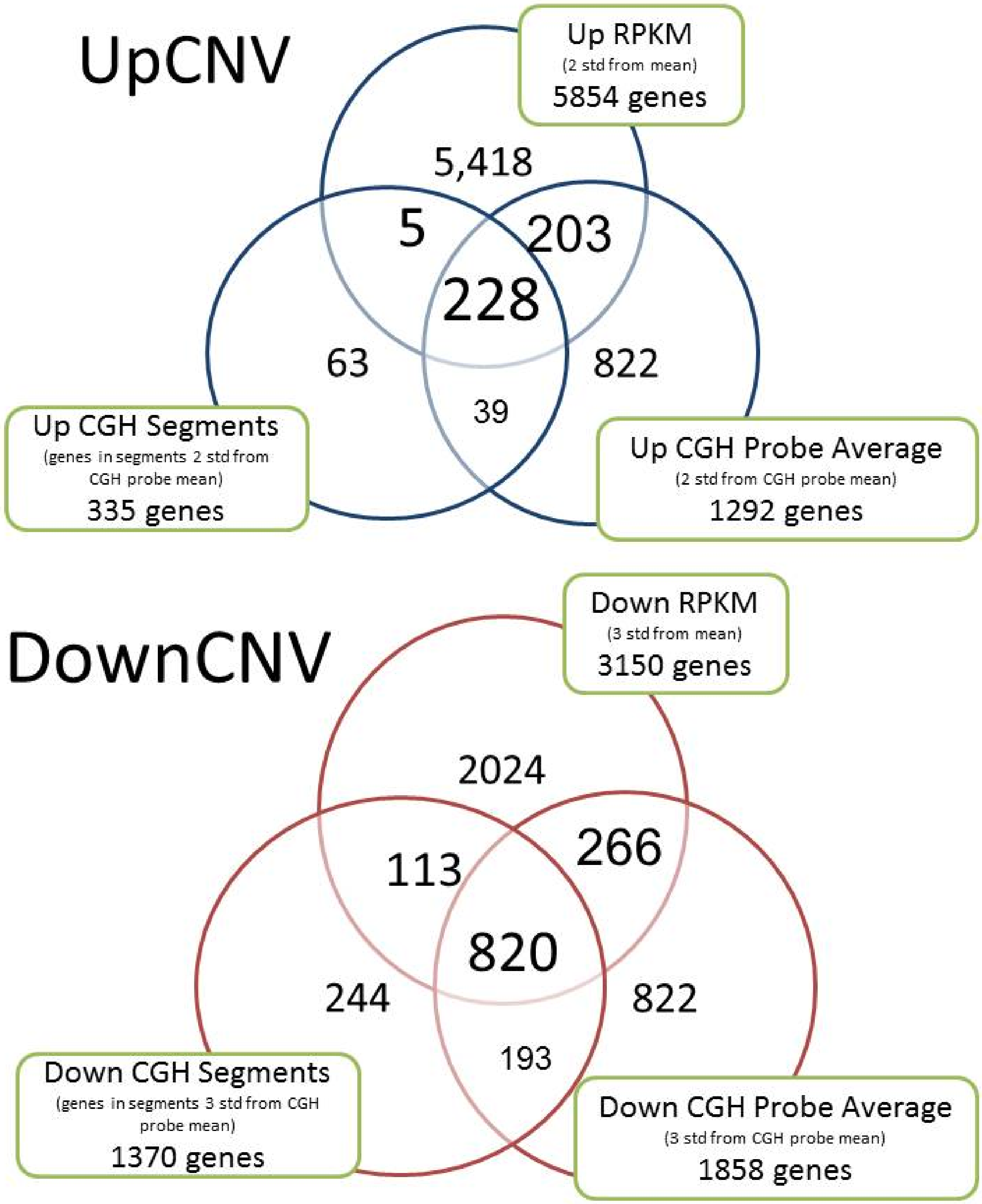

Figure S1 Venn diagram of the number of significant copy number variant gene models identified by three different detection methods (see Experimental procedures section for descriptions of the three methods).

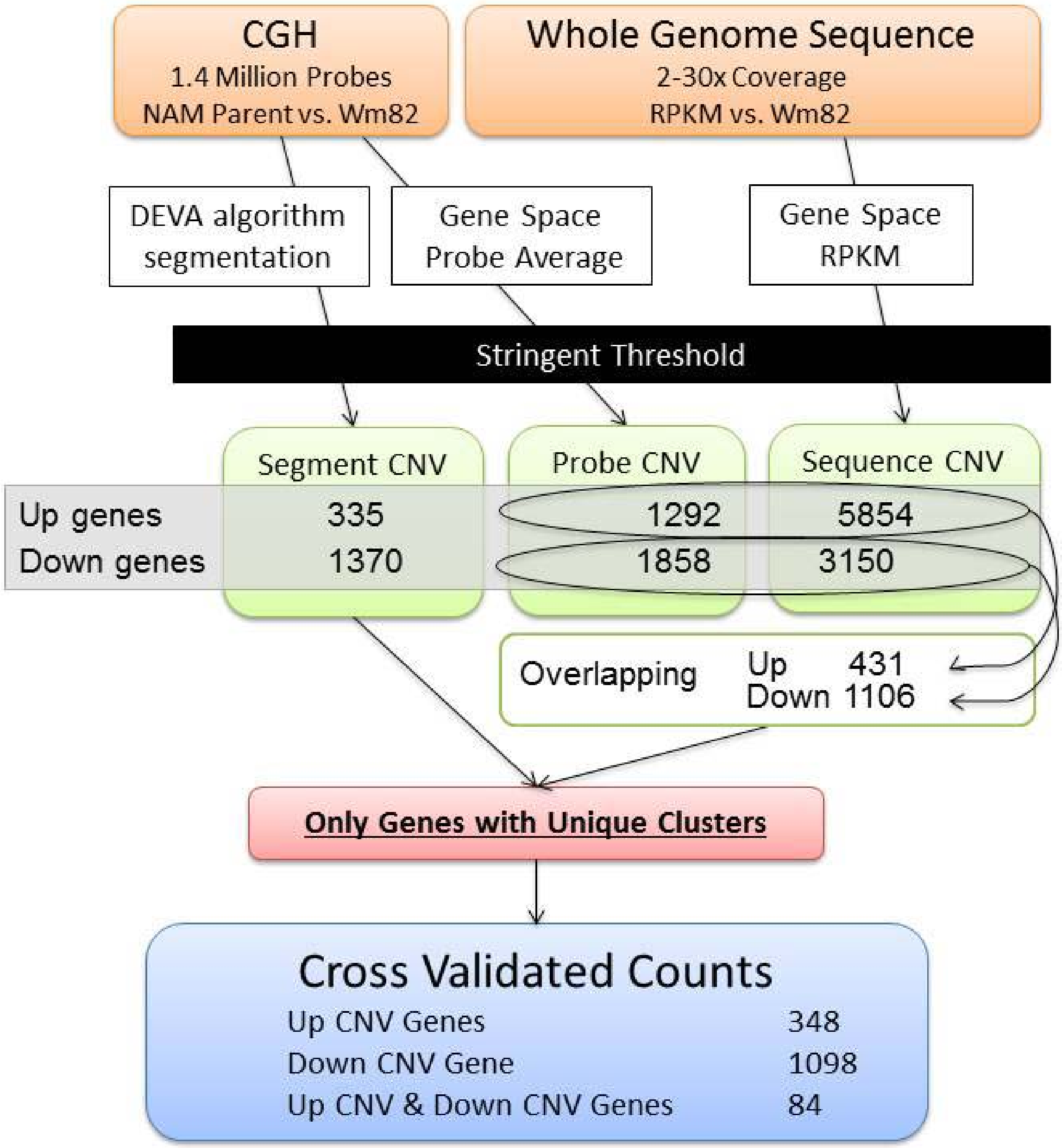

Figure S2 Methodological flow chart of the two data types and three different methods used in this analysis.

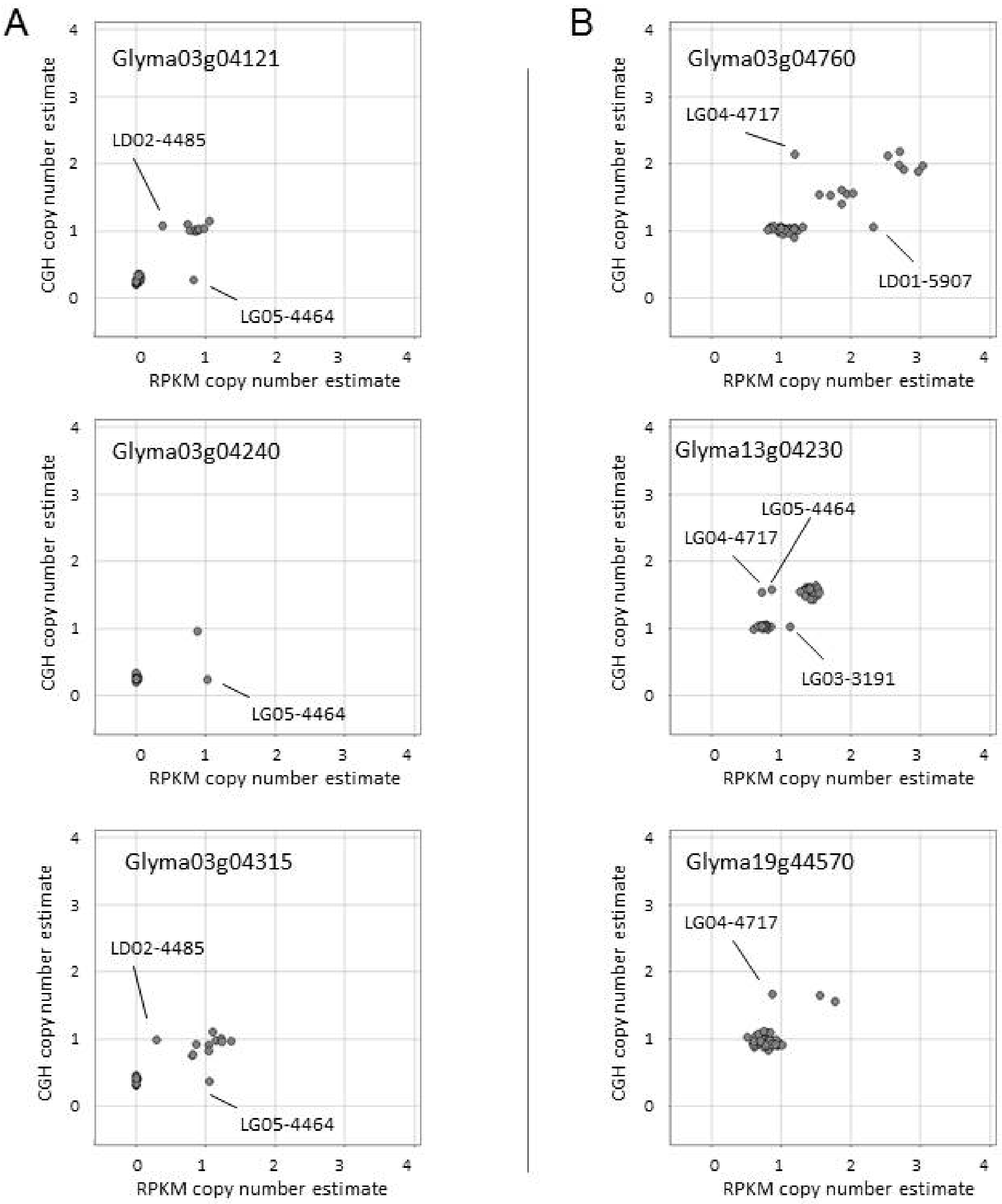

Figure S3 Presumed intra-cultivar heterogeneity results in incongruity between resequencing (x-axis) and CGH (y-axis) copy number estimates for some gene x cultivar comparisons. A) Line LG05-4464 (parent to the NAM 29 population) appears to be present in the resequencing data but absent in the CGH data across three neighboring genes. Presumably this is caused by heterogeneity between the two different individuals of LG05-4464 that were sampled for use with the respective platforms. The three genes shown in (A) are located within a 13 gene cluster that exhibits this presence-absence pattern for this genotype. (Also note that line LD02-4485 shows the opposite profile (absent-present) for two of the three genes, presumably also caused by intra-cultivar plant heterogeneity in this region.) B) Some lines exhibited recurrent incongruities throughout different regions of the genome, such as the single copy versus UpCNV patterns shown in (B) for line LG04-4717 (parent to the NAM 26 population) across three unlinked genes.

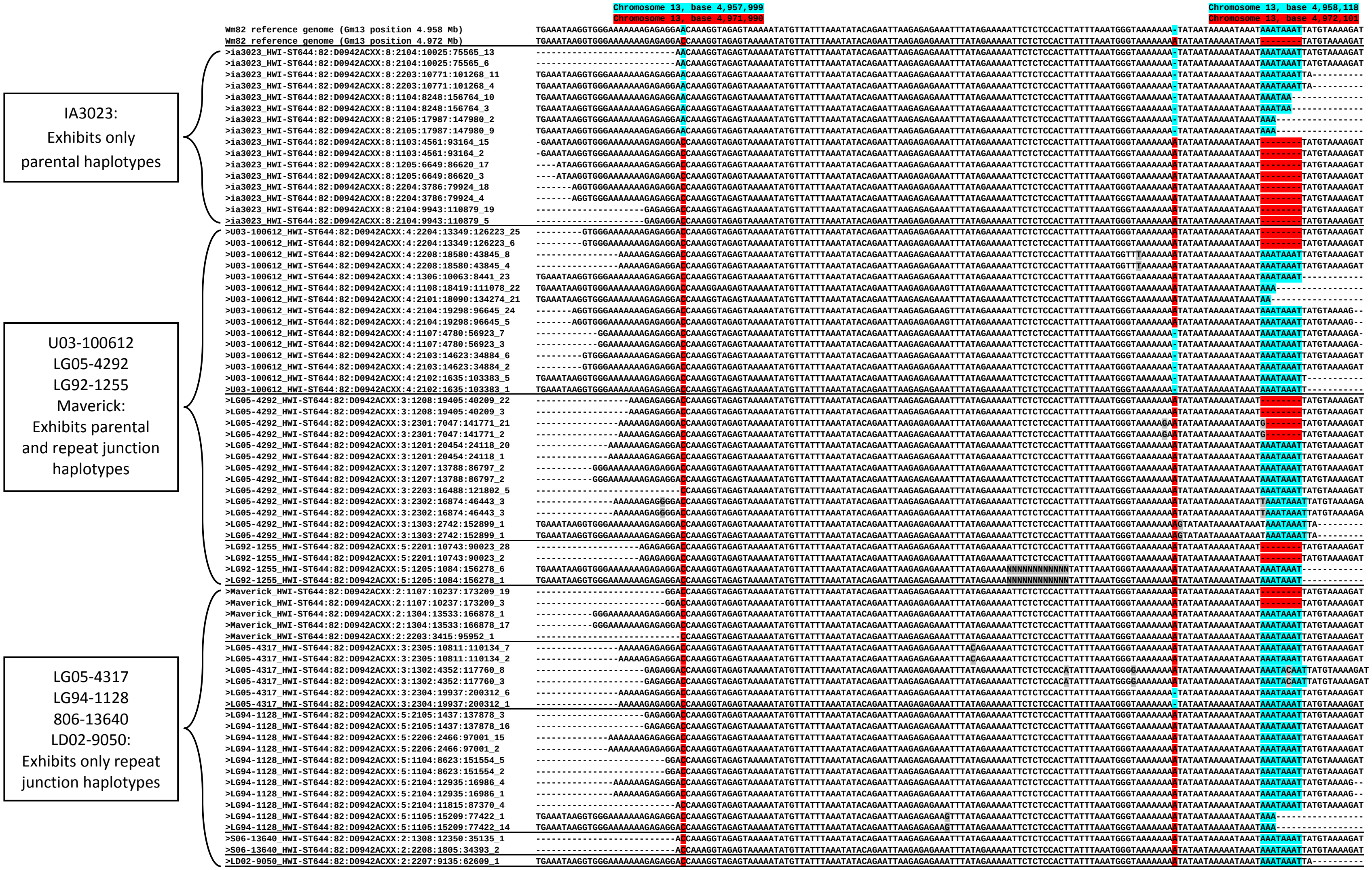

Figure S4 Sequence-based evidence for tandem repeats at a ~14-kb interval containing Glyma13g04670. The reference genome sequence of Wm82 exhibits two highly conserved regions, positioned at approximately 4.958 MB and 4.972 MB, respectively, on chromosome 13 (positions are from the version 1 genome assembly). The top two rows of the alignment show the similarity between these regions, with polymorphic bases shaded in blue or red, respectively. Sequence reads from IA3023, which CGH and resequencing analyses indicate have only one copy of Glyma13g04670, exhibits only the parental haplotype. For eight lines that CGH and resequencing analyses indicate have more than one copy of Glyma13g04670, reads are identified that start with the 4.972 MB haplotype and end with the 4.958 MB haplotype. This suggests that at least some of the amplification of the Glyma13g04670 gene is caused by tandem repeats of this ~14-kb unit.

A

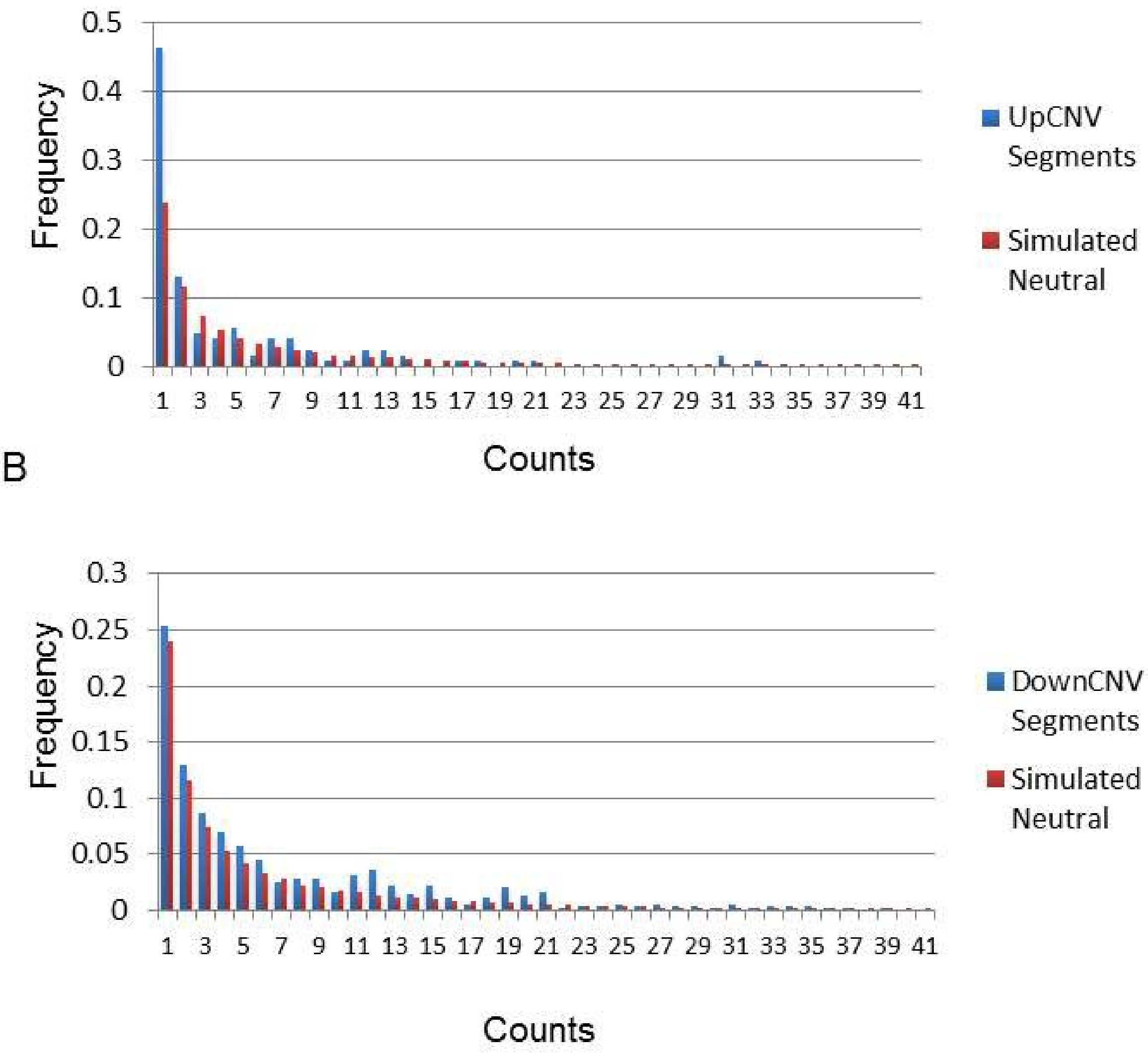

Figure S5 Reference based site frequency spectrum for UpCNV (A) and DownCNV (B) compared with simulated neutral frequencies. The frequency of singletons in the UpCNV class clearly exceeds the simulated neutral model while DownCNV frequencies follow the neutral simulation.

Table S1 Origin, maturity grouping and sequence depth of the soybean NAM parental genotypes assessed in this study (adapted from Stupar and Specht, 2013).

NAM Parent	Origin	NAM Population	Maturity	Sequence Coverage (Mapped Reads)	Estimated genome coverage
**IA3023**	Iowa State Univ.	Universal Parent	III	300645835	30.8
**TN05-3027**	Univ. of Tenn.	NAM 02	V	56222117	5.8
**4J105-3-4**	Purdue Univ.	NAM 03	III	61126856	6.3
**5M20-2-5-2**	Purdue Univ.	NAM 04	III	41247231	4.2
**CL0J095-4-6**	Purdue Univ.	NAM 05	III	48989486	5.0
**CL0J173-6-8**	Purdue Univ.	NAM 06	III	32994738	3.4
**HS6-3976**	Ohio State	NAM 08	III	43816809	4.5
**Prohio**	Ohio State Univ.	NAM 09	III	50197079	5.1
**LD00-3309**	Univ. of Illinois	NAM 10	IV	64726710	6.6
**LD01-5907**	Univ. of Illinois	NAM 11	IV	29287082	3.0
**LD02-4485**	Univ. of Illinois	NAM 12	III	38695512	4.0
**LD02-9050**	Univ. of Illinois	NAM 13	IV	31217207	3.2
**Magellan**	Univ. of Missouri	NAM 14	IV	19442222	2.0
**Maverick**	Univ. of Missouri	NAM 15	IV	29424567	3.0
**S06-13640**	Univ. of Missouri	NAM 17	IV	28792173	3.0
**NE3001**	Univ. of Nebraska	NAM 18	III	43531838	4.5
**Skylla**	Mich. State Univ.	NAM 22	III	34414411	3.5
**U03-100612**	Univ. of Nebraska	NAM 23	II	75305879	7.7
**LG03-2979**	USDA-ARS	NAM 24	III	69257822	7.1
**LG03-3191**	USDA-ARS	NAM 25	IV	59971468	6.2
**LG04-4717**	USDA-ARS	NAM 26	III	69257822	7.1
**LG05-4292**	USDA-ARS	NAM 27	IV	56823041	5.8
**LG05-4317**	USDA-ARS	NAM 28	IV	45144887	4.6
**LG05-4464**	USDA-ARS	NAM 29	III	47400111	4.9
**LG05-4832**	USDA-ARS	NAM 30	III	47797640	4.9
**LG90-2550**	USDA-ARS	NAM 31	III	26961469	2.8
**LG92-1255**	USDA-ARS	NAM 32	II	46875996	4.8
**LG94-1128**	USDA-ARS	NAM 33	II	29801583	3.1
**LG94-1906**	USDA-ARS	NAM 34	II	41179654	4.2
**LG97-7012**	USDA-ARS	NAM 36	III	29199662	3.0
**LG98-1605**	USDA-ARS	NAM 37	III	26685064	2.7
**LG00-3372**	USDA-ARS	NAM 38	III	35159242	3.6
**LG04-6000**	USDA-ARS	NAM 39	IV	34338365	3.5
**PI 398.881**	South Korea	NAM 40	III	48432038	5.0
**PI 427.136**	South Korea	NAM 41	III	61985465	6.4
**PI 437.169B**	Russia	NAM 42	II	66818192	6.9
**PI 507.681B**		NAM 46	II	50931853	5.2
**PI 518.751**	Serbia	NAM 48	II	72854403	7.5
**PI 561.370**	China	NAM 50	III	67853683	7.0
**PI 404.188A**	China	NAM 54	II	57653141	5.9
**PI 574.486**	China	NAM 64	II	36042594	3.7
**Williams 82-ISU-01**		Reference Genome	III	133013600	13.6

Table S2 Repeatability of technical replications at variable thresholds.

	Threshold log_2 ratio mean		Genes in Significant Down Segments				All probes in Significant Segments			
			Genes Found Significant		Significant Genes		Probes found significant		Significant Probes	
**Threshold**	Rep 1	Rep 2	Rep 1	Rep 2	Shared	Repeatability	Rep 1	Rep 2	Shared	Repeatability
**90%**	-0.196	-0.198	1496	1203	991	0.58	62268	51999	44660	0.64
**95%**	-0.253	-0.253	443	443	370	0.72	40382	34820	29702	0.65
**99%**	-0.433	-0.422	952	798	665	0.61	19715	19382	16378	0.72
**2 standard deviations**	-0.615	-0.575	341	365	291	0.70	14477	15485	12293	0.70
**3 standard deviations**	-0.922	-0.863	204	222	184	0.76	8850	8959	7638	0.75
**4 standard deviations**	-1.229	-1.151	164	128	111	0.61	6126	5483	4746	0.69

Tables S3-S6

Available for download as Excel files at http://www.g3journal.org/lookup/suppl/doi:10.1534/g3.114.011551/-/DC1

Table S3 Segmentation and Frequency of Up rSFS.

Table S4 Segmentation and Frequency of Down rSFS.

Table S5 Genes present in Cross-validated classes.

Table S6 Paralogous gene pairs in the soybean genome derived from ancient whole-genome duplication(s).
