# Supplementary material for: A Roadmap for Functional Structural Variants in the Soybean Genome
Source: G3 (Bethesda). 2014 May 22;4(7):1307–18. doi: 10.1534/g3.114.011551 (PMC4455779; doi:10.1534/g3.114.011551)
Supplement: Supporting Information [file supp_g3.114.011551_FileS1.pdf]

File S1

Determination of CGH thresholds

Probes on the microarray were designed to bind at unique single copy sequences in the reference genome. This assumption influenced the method used to determine significant segments in the CGH data. Applying this assumption, significant DownCNV should often represent sequences that are present as a single copy in Wm82-ISU-01, but are absent in the test genotype. However, it is also possible that hybridization differences can be caused by present but highly polymorphic sequences, which may also reduce the Cy3 signal from the test genotype. True PAV segments, in contrast, should exhibit a stronger $\log_2$ ratio reduction, as the denominator in the calculation will be expected to be nearly zero. Therefore, we applied a stringent threshold of three standard deviations to buffer against the detection of polymorphic sequences, and enrich the percentage of true PAV among the Down calls. Analysis of technical replicates of the IA3023 versus Wm82-ISU-01 comparison confirmed the highest level of repeatability using this threshold (Supplemental Table 4).

UpCNV threshold determination required a different set of assumptions. Again, segments were expected to be present as a single copy in Wm82-ISU-01. Furthermore, segments that were absent in Wm82-ISU-01 and present in the test genotype will exhibit large $\log_2$ ratio values that will most certainly exceed the threshold. Instead, the challenge is to detect the quantitative variants that are present as a single copy in Wm82-ISU-01 but present in two or three copies in the test genotype. The *Rhg1* locus, which harbors a well-defined copy number increase across a 31.2-kb interval on chromosome 18 (Cook *et al.* 2012), was used to empirically determine an appropriate threshold to accurately call UpCNV. It was determined that a threshold of two standard deviations above the mean was capable of detecting the 3-copy haplotype of *Rhg1*, whereas a three standard deviation threshold was not. Therefore, the two standard deviation threshold was applied across the samples for the detection of UpCNV segments.
