# Supplementary material for: A Roadmap for Functional Structural Variants in the Soybean Genome
Source: G3 (Bethesda). 2014 May 22;4(7):1307–18. doi: 10.1534/g3.114.011551 (PMC4455779; doi:10.1534/g3.114.011551)
Supplement: Supporting Information [file supp_g3.114.011551_FigureS1.pdf]

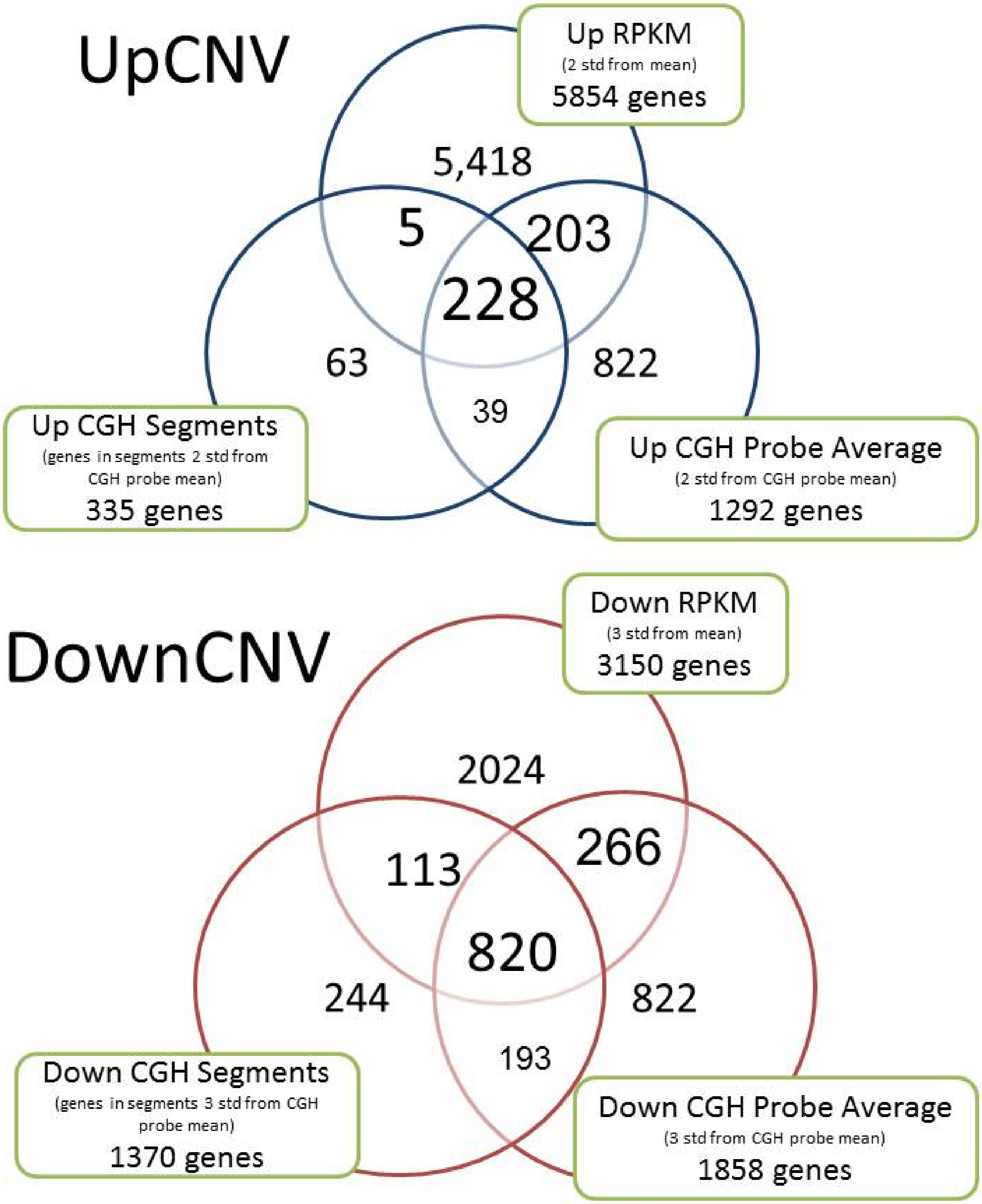

Figure S1 Venn diagram of the number of significant copy number variant gene models identified by three different detection methods (see Experimental procedures section for descriptions of the three methods).
