# Supplementary material for: A Roadmap for Functional Structural Variants in the Soybean Genome
Source: G3 (Bethesda). 2014 May 22;4(7):1307–18. doi: 10.1534/g3.114.011551 (PMC4455779; doi:10.1534/g3.114.011551)
Supplement: Supporting Information [file supp_g3.114.011551_FigureS3.pdf]

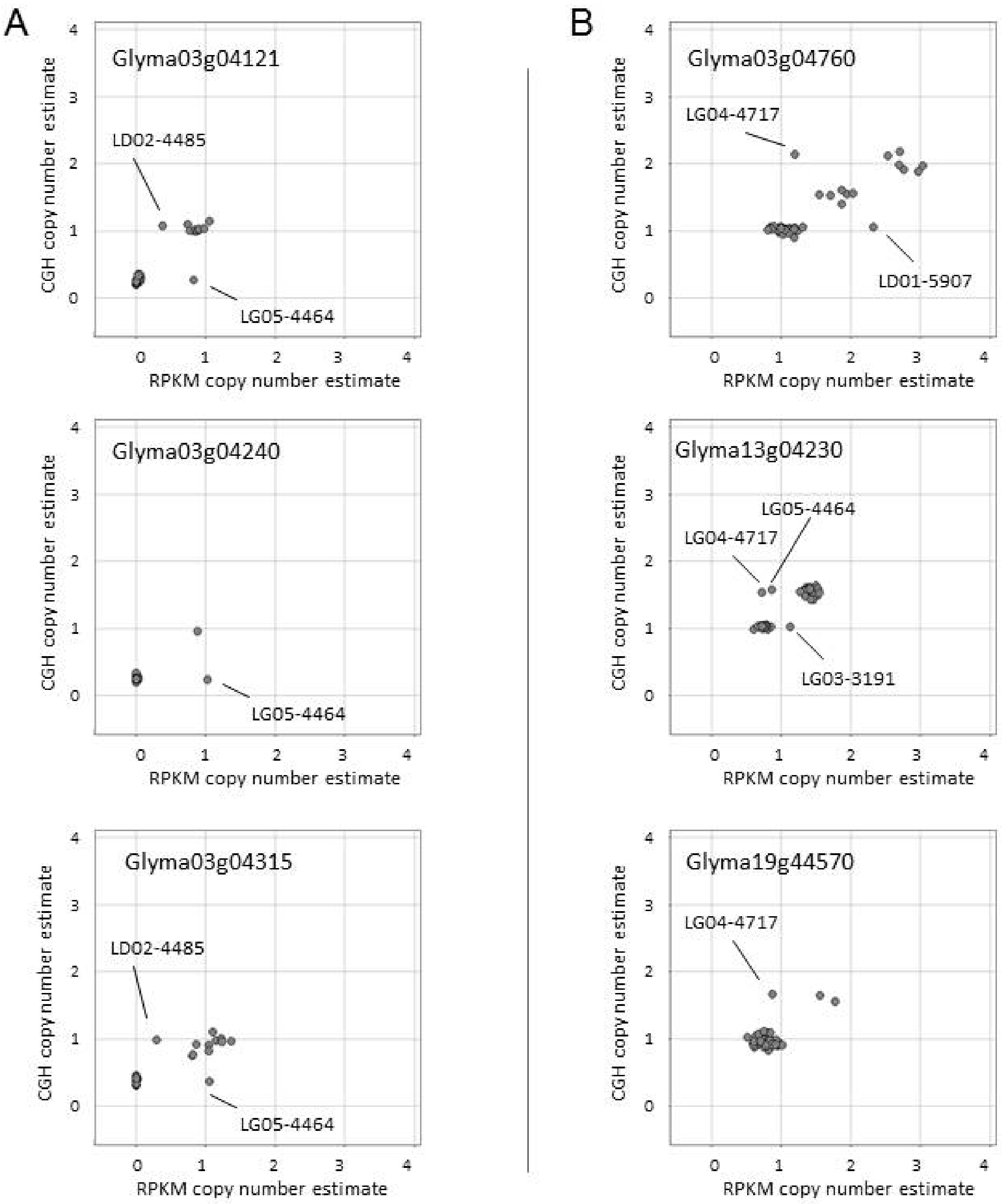

Figure S3 Presumed intra-cultivar heterogeneity results in incongruity between resequencing (x-axis) and CGH (y-axis) copy number estimates for some gene x cultivar comparisons. A) Line LG05-4464 (parent to the NAM 29 population) appears to be present in the resequencing data but absent in the CGH data across three neighboring genes. Presumably this is caused by heterogeneity between the two different individuals of LG05-4464 that were sampled for use with the respective platforms. The three genes shown in (A) are located within a 13 gene cluster that exhibits this presence-absence pattern for this genotype. (Also note that line LD02-4485 shows the opposite profile (absent-present) for two of the three genes, presumably also caused by intra-cultivar plant heterogeneity in this region.) B) Some lines exhibited recurrent incongruities throughout different regions of the genome, such as the single copy versus UpCNV patterns shown in (B) for line LG04-4717 (parent to the NAM 26 population) across three unlinked genes.
