# Supplementary material for: A Roadmap for Functional Structural Variants in the Soybean Genome
Source: G3 (Bethesda). 2014 May 22;4(7):1307–18. doi: 10.1534/g3.114.011551 (PMC4455779; doi:10.1534/g3.114.011551)
Supplement: Supporting Information [file supp_g3.114.011551_FigureS4.pdf]

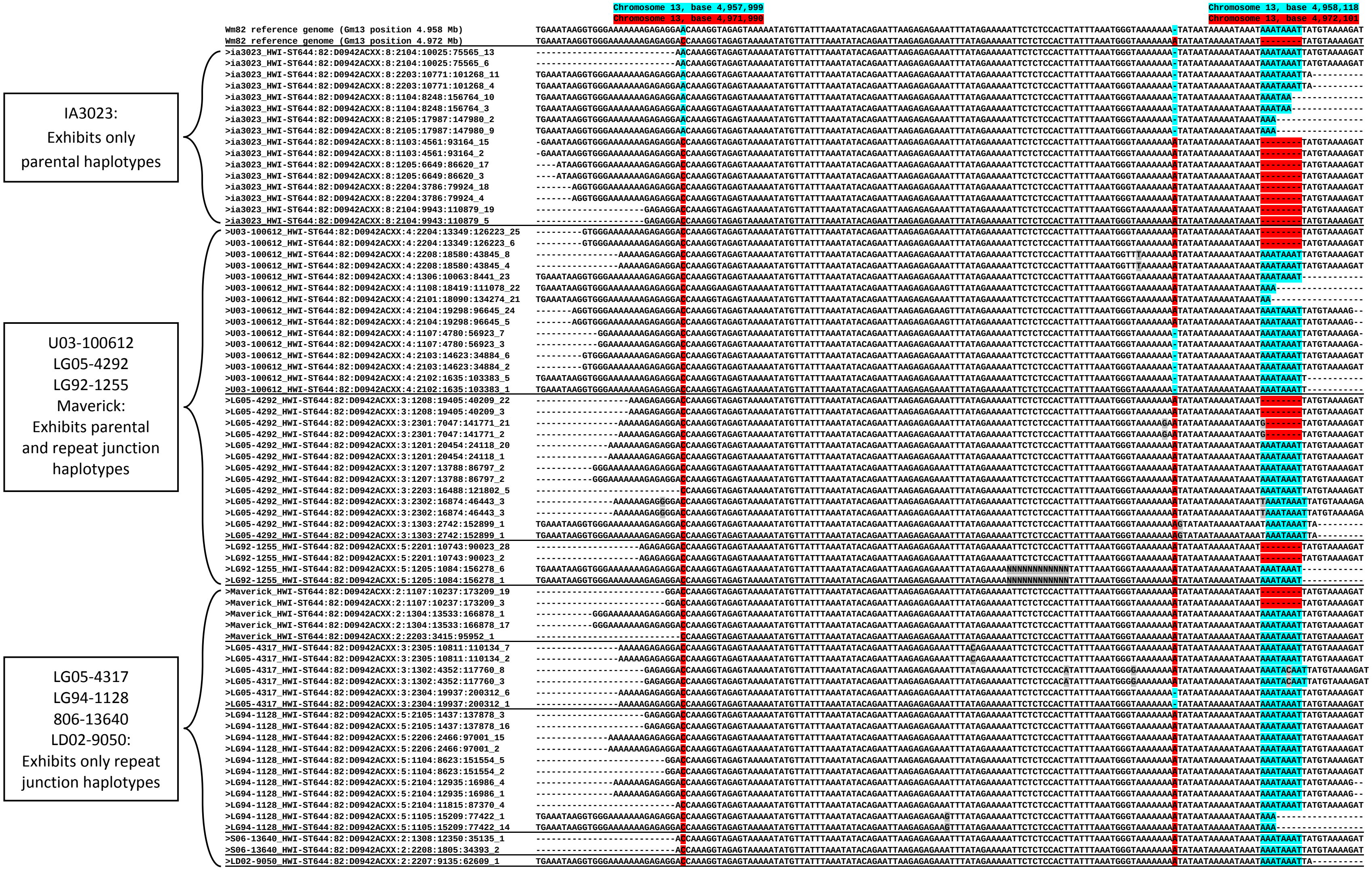

Figure S4 Sequence-based evidence for tandem repeats at a ~14-kb interval containing Glyma13g04670. The reference genome sequence of Wm82 exhibits two highly conserved regions, positioned at approximately 4.958 MB and 4.972 MB, respectively, on chromosome 13 (positions are from the version 1 genome assembly). The top two rows of the alignment show the similarity between these regions, with polymorphic bases shaded in blue or red, respectively. Sequence reads from IA3023, which CGH and resequencing analyses indicate have only one copy of Glyma13g04670, exhibits only the parental haplotype. For eight lines that CGH and resequencing analyses indicate have more than one copy of Glyma13g04670, reads are identified that start with the 4.972 MB haplotype and end with the 4.958 MB haplotype. This suggests that at least some of the amplification of the Glyma13g04670 gene is caused by tandem repeats of this ~14-kb unit.
