# Supplementary material for: A Roadmap for Functional Structural Variants in the Soybean Genome
Source: G3 (Bethesda). 2014 May 22;4(7):1307–18. doi: 10.1534/g3.114.011551 (PMC4455779; doi:10.1534/g3.114.011551)
Supplement: Supporting Information [file supp_g3.114.011551_FigureS5.pdf]

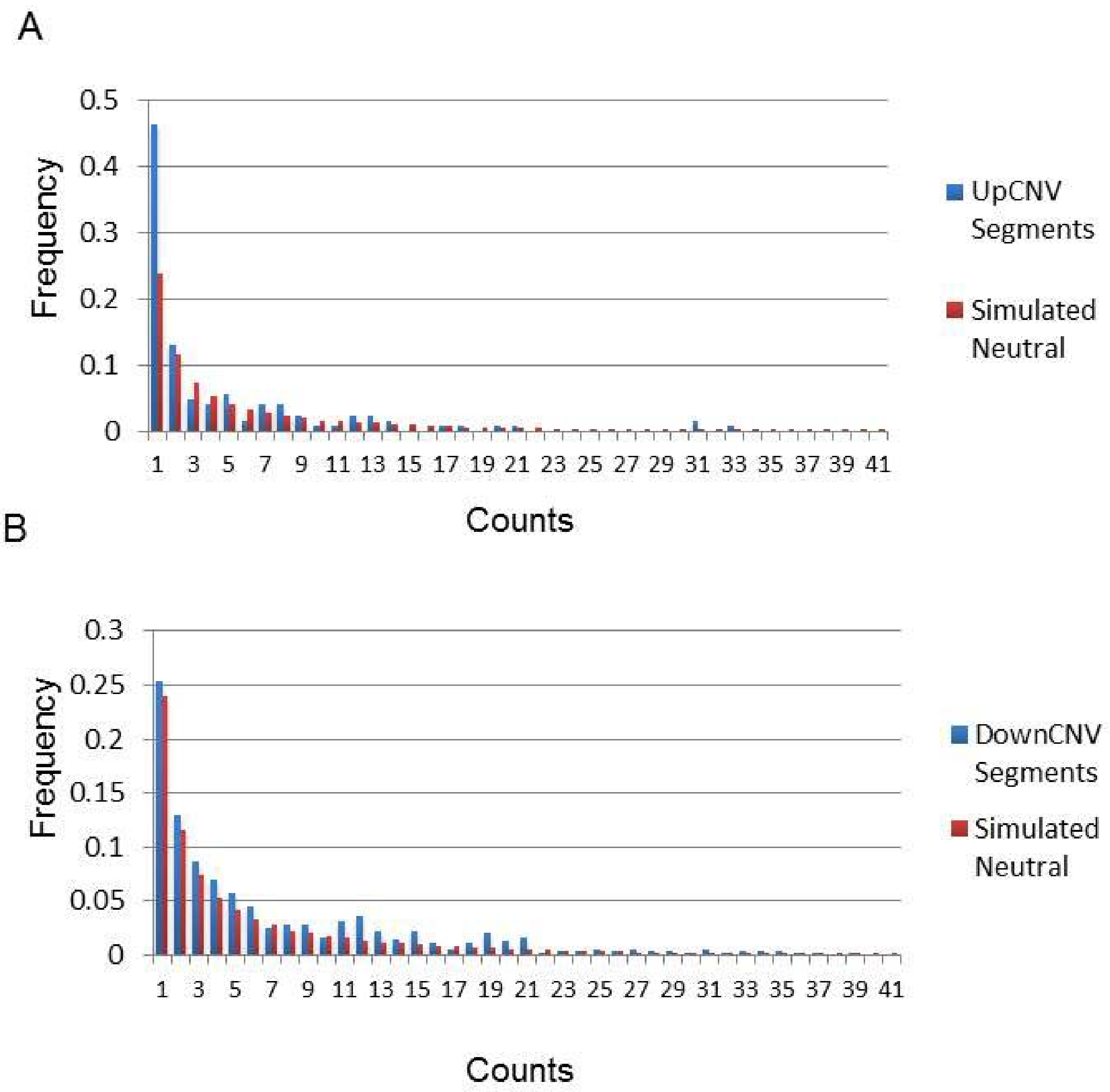

Figure S5 Reference based site frequency spectrum for UpCNV (A) and DownCNV (B) compared with simulated neutral frequencies. The frequency of singletons in the UpCNV class clearly exceeds the simulated neutral model while DownCNV frequencies follow the neutral simulation.
