# Supplementary material for: A Roadmap for Functional Structural Variants in the Soybean Genome
Source: G3 (Bethesda). 2014 May 22;4(7):1307–18. doi: 10.1534/g3.114.011551 (PMC4455779; doi:10.1534/g3.114.011551)
Supplement: Supporting Information [file supp_g3.114.011551_TableS1.pdf]

Table S1 Origin, maturity grouping and sequence depth of the soybean NAM parental genotypes assessed in this study (adapted from Stupar and Specht, 2013).

NAM Parent	Origin	NAM Population	Maturity	Sequence Coverage (Mapped Reads)	Estimated genome coverage
**IA3023**	Iowa State Univ.	Universal Parent	III	300645835	30.8
**TN05-3027**	Univ. of Tenn.	NAM 02	V	56222117	5.8
**4J105-3-4**	Purdue Univ.	NAM 03	III	61126856	6.3
**5M20-2-5-2**	Purdue Univ.	NAM 04	III	41247231	4.2
**CL0J095-4-6**	Purdue Univ.	NAM 05	III	48989486	5.0
**CL0J173-6-8**	Purdue Univ.	NAM 06	III	32994738	3.4
**HS6-3976**	Ohio State	NAM 08	III	43816809	4.5
**Prohio**	Ohio State Univ.	NAM 09	III	50197079	5.1
**LD00-3309**	Univ. of Illinois	NAM 10	IV	64726710	6.6
**LD01-5907**	Univ. of Illinois	NAM 11	IV	29287082	3.0
**LD02-4485**	Univ. of Illinois	NAM 12	III	38695512	4.0
**LD02-9050**	Univ. of Illinois	NAM 13	IV	31217207	3.2
**Magellan**	Univ. of Missouri	NAM 14	IV	19442222	2.0
**Maverick**	Univ. of Missouri	NAM 15	IV	29424567	3.0
**S06-13640**	Univ. of Missouri	NAM 17	IV	28792173	3.0
**NE3001**	Univ. of Nebraska	NAM 18	III	43531838	4.5
**Skylla**	Mich. State Univ.	NAM 22	III	34414411	3.5
**U03-100612**	Univ. of Nebraska	NAM 23	II	75305879	7.7
**LG03-2979**	USDA-ARS	NAM 24	III	69257822	7.1
**LG03-3191**	USDA-ARS	NAM 25	IV	59971468	6.2
**LG04-4717**	USDA-ARS	NAM 26	III	69257822	7.1
**LG05-4292**	USDA-ARS	NAM 27	IV	56823041	5.8
**LG05-4317**	USDA-ARS	NAM 28	IV	45144887	4.6
**LG05-4464**	USDA-ARS	NAM 29	III	47400111	4.9
**LG05-4832**	USDA-ARS	NAM 30	III	47797640	4.9
**LG90-2550**	USDA-ARS	NAM 31	III	26961469	2.8
**LG92-1255**	USDA-ARS	NAM 32	II	46875996	4.8
**LG94-1128**	USDA-ARS	NAM 33	II	29801583	3.1
**LG94-1906**	USDA-ARS	NAM 34	II	41179654	4.2
**LG97-7012**	USDA-ARS	NAM 36	III	29199662	3.0
**LG98-1605**	USDA-ARS	NAM 37	III	26685064	2.7
**LG00-3372**	USDA-ARS	NAM 38	III	35159242	3.6
**LG04-6000**	USDA-ARS	NAM 39	IV	34338365	3.5
**PI 398.881**	South Korea	NAM 40	III	48432038	5.0
**PI 427.136**	South Korea	NAM 41	III	61985465	6.4
**PI 437.169B**	Russia	NAM 42	II	66818192	6.9
**PI 507.681B**		NAM 46	II	50931853	5.2
**PI 518.751**	Serbia	NAM 48	II	72854403	7.5
**PI 561.370**	China	NAM 50	III	67853683	7.0
**PI 404.188A**	China	NAM 54	II	57653141	5.9
**PI 574.486**	China	NAM 64	II	36042594	3.7
**Williams 82-ISU-01**		Reference Genome	III	133013600	13.6
