# Supplementary material for: A Roadmap for Functional Structural Variants in the Soybean Genome
Source: G3 (Bethesda). 2014 May 22;4(7):1307–18. doi: 10.1534/g3.114.011551 (PMC4455779; doi:10.1534/g3.114.011551)
Supplement: Supporting Information [file supp_g3.114.011551_TableS2.pdf]

Table S2 Repeatability of technical replications at variable thresholds.

Threshold	Threshold log_2 ratio mean		Genes in Significant Down Segments				All probes in Significant Segments			
			Genes Found Significant		Significant Genes Shared		Probes found significant		Significant Probes Shared	
	Rep 1	Rep 2	Rep 1	Rep 2		Repeatability	Rep 1	Rep 2		Repeatability
**90%**	-0.196	-0.198	1496	1203	991	0.58	62268	51999	44660	0.64
**95%**	-0.253	-0.253	443	443	370	0.72	40382	34820	29702	0.65
**99%**	-0.433	-0.422	952	798	665	0.61	19715	19382	16378	0.72
**2 standard deviations**	-0.615	-0.575	341	365	291	0.70	14477	15485	12293	0.70
**3 standard deviations**	-0.922	-0.863	204	222	184	0.76	8850	8959	7638	0.75
**4 standard deviations**	-1.229	-1.151	164	128	111	0.61	6126	5483	4746	0.69
